# GCL pruning of PIP3 establishes the soma-germline boundary

**DOI:** 10.1083/jcb.202604036

**Published:** 2026-07-22

**Authors:** Mariyah Saiduddin, Juhee Pae, Asier Marcos-Vidal, Martin L. Alani, Ruth Lehmann

**Affiliations:** 1 Whitehead Institute for Biomedical Research, Cambridge, MA, USA; 2 Vilcek Institute of Graduate Biomedical Sciences, NYU Grossman School of Medicine, New York, NY, USA; 3 Rockefeller University, New York, NY, USA; 4 Department of Biology, Massachusetts Institute of Technology, Cambridge, MA, USA

## Abstract

Primordial germ cells (PGCs) are the first cells specified in the *Drosophila* embryo and are precursors to the germline. Their formation requires suppression of somatic fates, achieved by degrading the receptor tyrosine kinase Torso at the posterior pole through the ubiquitin ligase adaptor germ cell-less (GCL). Although Torso is known to antagonize PGC formation, the underlying mechanisms remained unclear. Here, we combine optogenetic Ras activation and Ras effector loop mutants to show that Ras suppresses PGC formation independently of the canonical Raf/MEK/ERK pathway. We identify an unexpected early role for Torso in activating phosphoinositide 3-kinase (PI3K), generating membrane domains enriched in phosphatidylinositol (3,4,5)-trisphosphate (PIP3). Elevated PI3K activity disrupts PGC formation, while reduced PI3K activity creates ectopic PGCs. We demonstrate that GCL remodels the posterior pole membrane by suppressing Torso-dependent PI3K activation. Clearing PIP3 enables myosin II enrichment, allowing for PGC formation. Together, our findings reveal how antagonistic Torso and GCL activities establish the soma-germline boundary by organizing cortical lipids.

## Teaser

Saiduddin et al. use optogenetics and live imaging in *Drosophila* embryos to identify an unexpected role of Torso receptor tyrosine kinase in activating PI3K signaling. They demonstrate that the germplasm-specific ubiquitin ligase adaptor GCL is required to prune PIP3 from the membrane to establish the soma-germline boundary.

## Graphical Abstract

**Figure:**
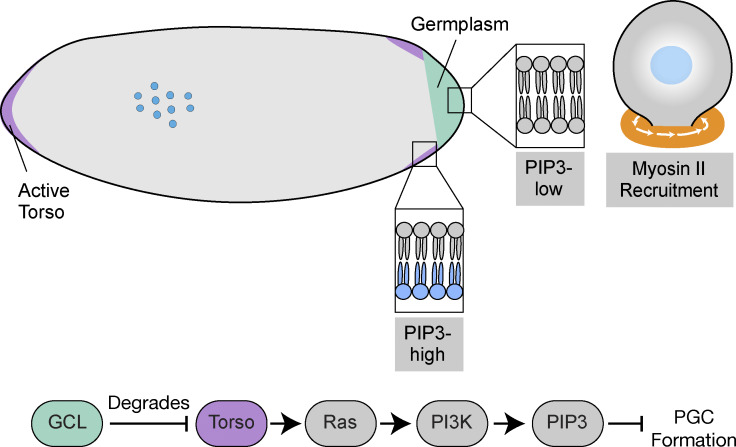

## Introduction

Most embryos develop from a large egg that, upon fertilization, is divided into cells. In most insects, cellularization is delayed, allowing the embryo to initiate embryogenesis in a syncytial phase, with nuclei dividing within a shared cytoplasm. This process has been studied most extensively in *Drosophila melanogaster*, where, after fertilization, nuclei undergo rapid synchronous divisions. After nine divisions, the nuclei migrate to the embryo’s cortex, where membrane remodeling leads to the formation of pseudocleavage furrows that bulge from the cortex (Foe and Alberts, 1983). Only at the posterior pole of the embryo do these buds form cells, while the remainder of the embryo continues syncytial divisions. This difference arises from the posterior enrichment of a specialized cytoplasmic compartment, the germplasm, which the mother deposits into the embryo during oogenesis. The maternally contributed germplasm contains all the factors necessary to form and specify the embryo’s first cells, pole cells or primordial germ cells (PGCs), which will develop into the germline in the resulting adult (Illmensee and Mahowald, 1974). Zygotic transcription is not essential for the initial formation of PGCs, making PGC formation an attractive model for studying early cell formation in other organisms that also depend on maternally provided factors (Cinalli and Lehmann, 2013). In contrast, somatic cells form after the 13th nuclear division in a specialized membrane-deposition process that requires new zygotic transcription by the embryo.

PGC formation occurs through two orthogonal constrictions: a “traditional” spindle-dependent anaphase furrow constriction that divides one pole bud into two, and a spindle-independent “bud furrow” constriction that pinches off the dividing pole buds from the rest of the embryo cytoplasm (Cinalli and Lehmann, 2013). Constriction of these two bud furrows must happen within a brief window during nuclear cycle 10 for PGCs to fully detach from the embryo, thereby protecting the integrity of the PGC-specifying germplasm (Lerit and Gavis, 2011). How these two constrictions are regulated and coordinated remains unclear. After both furrows constrict and PGCs form, germplasm and nuclei are entirely segregated from the rest of the soma, allowing PGC specification to proceed. Once encased in a membrane, PGCs divide independently of the syncytial nuclear divisions. They undergo up to two divisions until the end of nuclear cycle 14, when morphogenetic gastrulation movements initiate their migratory journey through the embryo toward the somatic gonad (Chen et al., 2025).

One component of germplasm, germ cell-less (GCL), is critical for the formation of PGCs (Jongens et al., 1994). Without maternally deposited *gcl* RNA and its localized translation at the posterior pole, embryos laid by *gcl−/−* mothers (heretofore referred to as *gcl−/−* embryos) fail to undergo bud furrow constriction by the end of nuclear cycle 10, although anaphase furrow constrictions continue. As a result, pole buds fail to cellularize, and because germplasm is not properly sequestered and protected in PGCs, it is degraded (Iida and Kobayashi, 2000). A minority of *gcl−/−* embryos (<20%) will form PGCs, though with greatly reduced numbers. GCL encodes a ubiquitin ligase adaptor, and the receptor tyrosine kinase (RTK) Torso was identified as its specific substrate for degradation (Pae et al., 2017). Torso’s RTK activity is restricted to the anterior and posterior regions of the developing oocyte due to the spatially regulated processing of its ligand, Trunk (Sprenger and Nüsslein-Volhard, 1992; Stevens et al., 1990). Through GCL-mediated degradation of Torso, RTK activity is absent from the pole buds at the posterior pole. Indeed, Torso’s depletion at the posterior pole is necessary for PGCs to form, as the *gcl−/−* PGC defect can be fully rescued by preventing Torso’s RTK activity (Pae et al., 2017). Therefore, GCL shields posterior pole buds from RTK interference as they cellularize, allowing a parallel pathway, regulated by Rho1, anillin, and other components of the contractile ring, to direct pole bud cellularization (Field et al., 2005; Padash Barmchi et al., 2005).

It remains unclear how Torso RTK antagonizes PGC formation. Torso’s signaling role in embryogenesis activates the Ras/Raf/MEK/ERK pathway and triggers the transcription of patterning genes, such as the *huckebein* and *tailless* transcription factors, which are necessary for developing the embryonic termini (Brönner and Jäckle, 1991). However, several observations argue against a transcriptional role for Torso in PGC formation: First, the embryo has not fully activated its zygotic transcription and relies primarily on maternally deposited factors at the time of PGC formation. Second, if transcription needed to be suppressed to allow for PGC formation, one would expect that inhibiting RNA Polymerase II would rescue the *gcl−/−* phenotype, but it does not (Cinalli and Lehmann, 2013). Finally, the *gcl−/−* mutant phenotype can be rescued by knocking down the ArfGEF Steppke (Lee et al., 2015), a regulator of endocytosis, suggesting that inappropriate activation of Torso affects cytoskeletal organization rather than transcription.

We investigated Torso signaling to identify downstream effectors that interfere with PGC formation. Our results demonstrate a role for Torso signaling at the onset of embryonic development, independent of its known, later function in the transcriptional activation of terminal patterning genes. We identify PI3K as a critical downstream component of Torso activation and reveal an unexpected role for this signaling pathway early in embryogenesis in defining the germline-soma boundary via lipid domains.

## Results

### Light-induced activation of Ras causes failure in PGC formation

**Figure 1.**
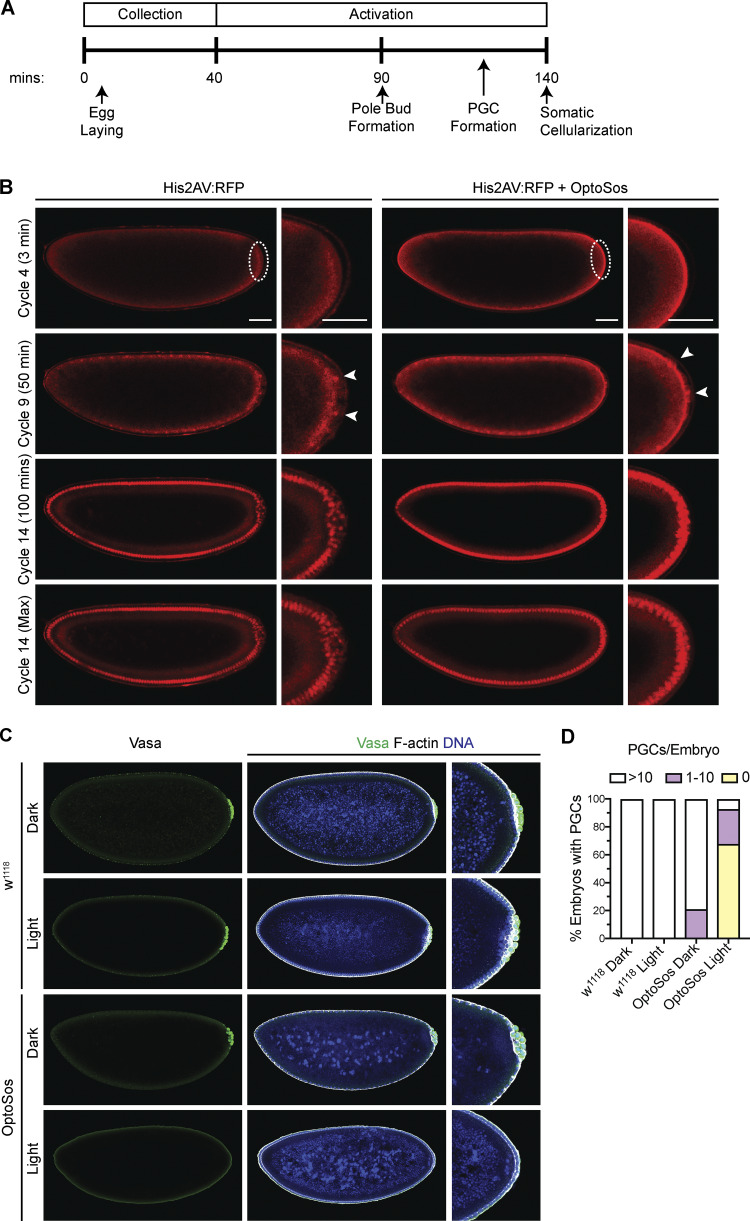
**The RasGEF activity of Sos is sufficient to inhibit PGC formation, but not pole bud formation. (A)** Timeline for optogenetic Ras activation. **(B)** Embryos from mothers expressing His2AV:RFP alone or His2AV:RFP together with OptoSos were imaged under blue-light activation in a region of interest (outlined with a dotted white oval). Arrowheads indicate the pole bud nuclei that protrude against the posterior membrane. In embryos from mothers that express His2AV:RFP alone, a group of rounded nuclei was observed adjacent to the layer of elongated somatic nuclei. This group of rounded nuclei was not observed in embryos from mothers expressing both His2AV:RFP and the OptoSos construct. The first three rows show z-plane images, while the bottom row shows a maximum-intensity projection. Embryos are cycle 9–10. Scale bar = 50 μm. **(C)** Representative embryo for each indicated condition. Fixed embryos were immunostained with anti-Vasa (Green). F-actin (gray) outlines the membrane. DNA (blue). **(D)** Embryos from WT (*w*^*1118*^) or OptoSos-expressing mothers were either kept in the dark (dark) or activated with blue light at the posterior pole (light). After the 100-min activation cycle, embryos were fixed and stained with an antibody against Vasa to assess PGC formation. The percentage of embryos with the indicated number of PGCs was scored. *N* = 20 embryos for *w*^*1118*^ dark, *n* = 14 embryos for *w*^*1118*^ light, *n* = 12 embryos for OptoSos dark, and *n* = 20 embryos for OptoSos light.

Previous studies indicated that ectopic activation of the Torso RTK pathway represses the cellular processes leading to PGC formation (Pae et al., 2017). As the canonical Torso RTK pathway recruits adaptor molecules to the membrane that activate Ras (Li, 2005), we aimed to determine whether Ras activation is sufficient to interfere with PGC formation. Therefore, we employed an optogenetic approach, OptoSos, which allows for precise spatiotemporal activation of Ras, independent of RTK activity (Johnson et al., 2017). This is achieved by blue light-induced translocation of Sos, a Ras guanine nucleotide exchange factor (GEF), to the plasma membrane (OptoSos) (Toettcher et al., 2013; Johnson et al., 2017). This precise spatiotemporal control of Ras activation has two major advantages: (1) we could activate Ras specifically at the posterior pole while allowing the rest of the embryo to develop normally; and (2) we could induce ectopic signaling at a developmental stage before the Torso receptor activates embryonic somatic patterning (Casanova and Struhl, 1989). Thus, instead of Ras activation at the blastoderm stage when PGCs had already formed (Johnson et al., 2017), we exposed the posterior pole of embryos to blue light at the syncytial stage prior to PGC formation (starting at 40 min after egg deposition) (Fig. 1 A). To visualize the developmental process during time-lapse imaging, we also expressed the nuclear marker His2AV:RFP. Live imaging showed that pole buds still formed and protruded from the embryo cortex, but PGCs largely failed to cellularize, while control embryos that lacked the OptoSos blue light sensor formed PGCs normally (Fig. 1 B).

To quantitatively assess these live observations, embryos were immunostained with an antibody against Vasa to count PGCs at nuclear cycle 14, when PGC divisions had ceased (Fig. 1 C). 90 percent of embryos expressing OptoSos that were activated with blue light during the syncytial stage showed a reduction or complete loss of PGCs compared with control embryos that were kept in the dark (20% showed a reduced number of PGCs) or *w*^*1118*^ embryos that received either dark or light treatment (no reduction in PGCs) (Fig. 1 D). These data demonstrate that early activation of Ras is sufficient to prevent pole buds from completing cellularization, even in the presence of GCL.

### Torso activation interferes with PGC formation independently of the Raf–MEK–ERK pathway

**Figure 2.**
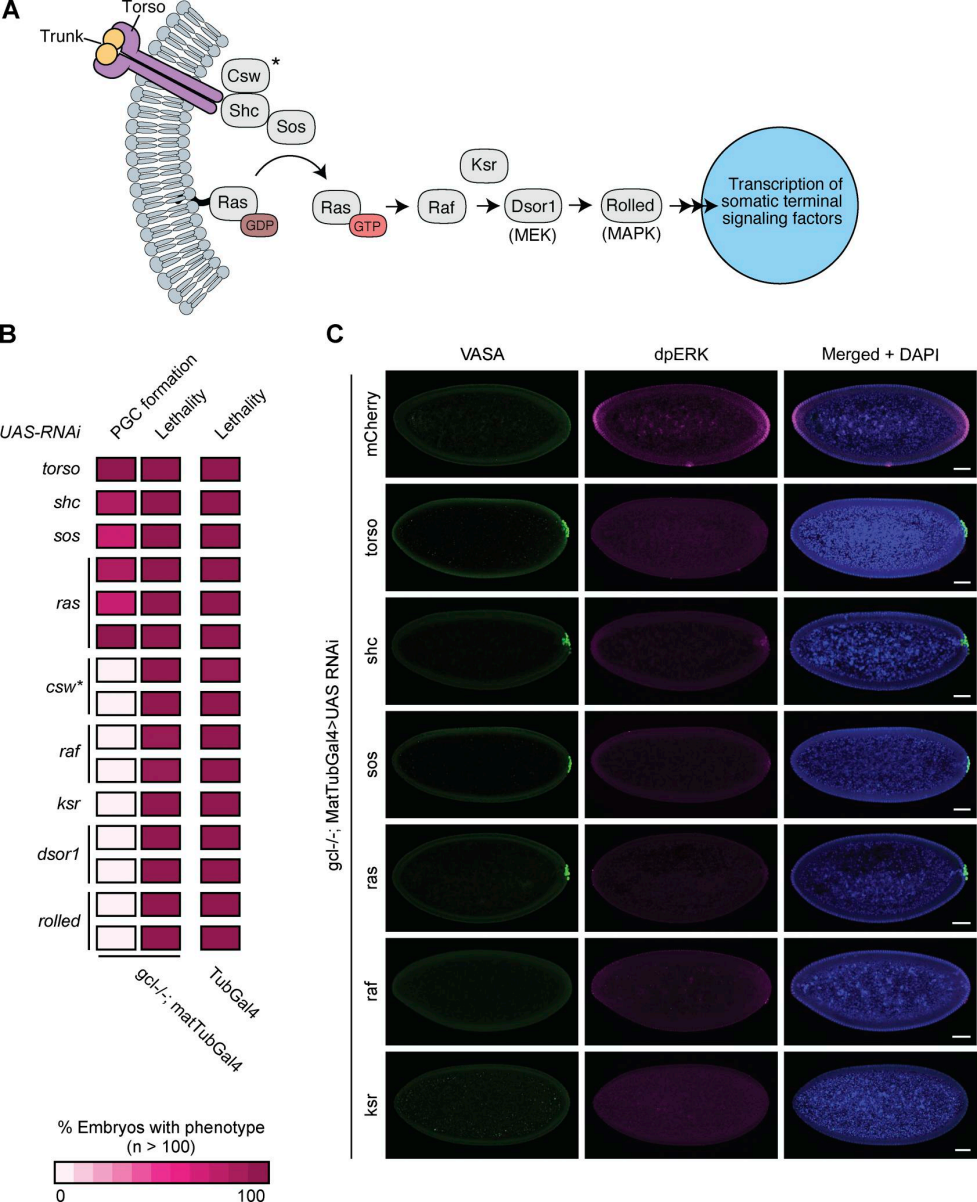
**Ras is the most downstream component in the canonical Torso signaling pathway that can antagonize PGC formation. (A)** Diagram of the canonical Torso signaling pathway, which is required to upregulate transcription of somatic patterning genes in the embryo. *Note that these results place Csw downstream of Ras, consistent with findings by Allard et al. (1996). **(B)** The canonical Torso signaling pathway components were knocked down in *gcl−/−* mothers, using RNAi driven with the germline-specific driver matTubGal4 during oogenesis. The percentage of embryos from these mutant mothers with Vasa-positive PGCs was calculated and plotted as a score for PGC formation. Lethality in these embryos was also scored. The ubiquitous driver TubGAL4 was used to assess knockdown efficiency. *n* > 100 embryos were scored for each condition. **(C)** Representative embryo of indicated maternal genotypes, nuclear cycle 13–14. Fixed embryos were immunostained with anti-Vasa (green) to count PGCs and anti-dpERK (magenta) to assess knock-down efficiency. DNA (blue). Scale bar = 50 μm.

**Figure S1.**
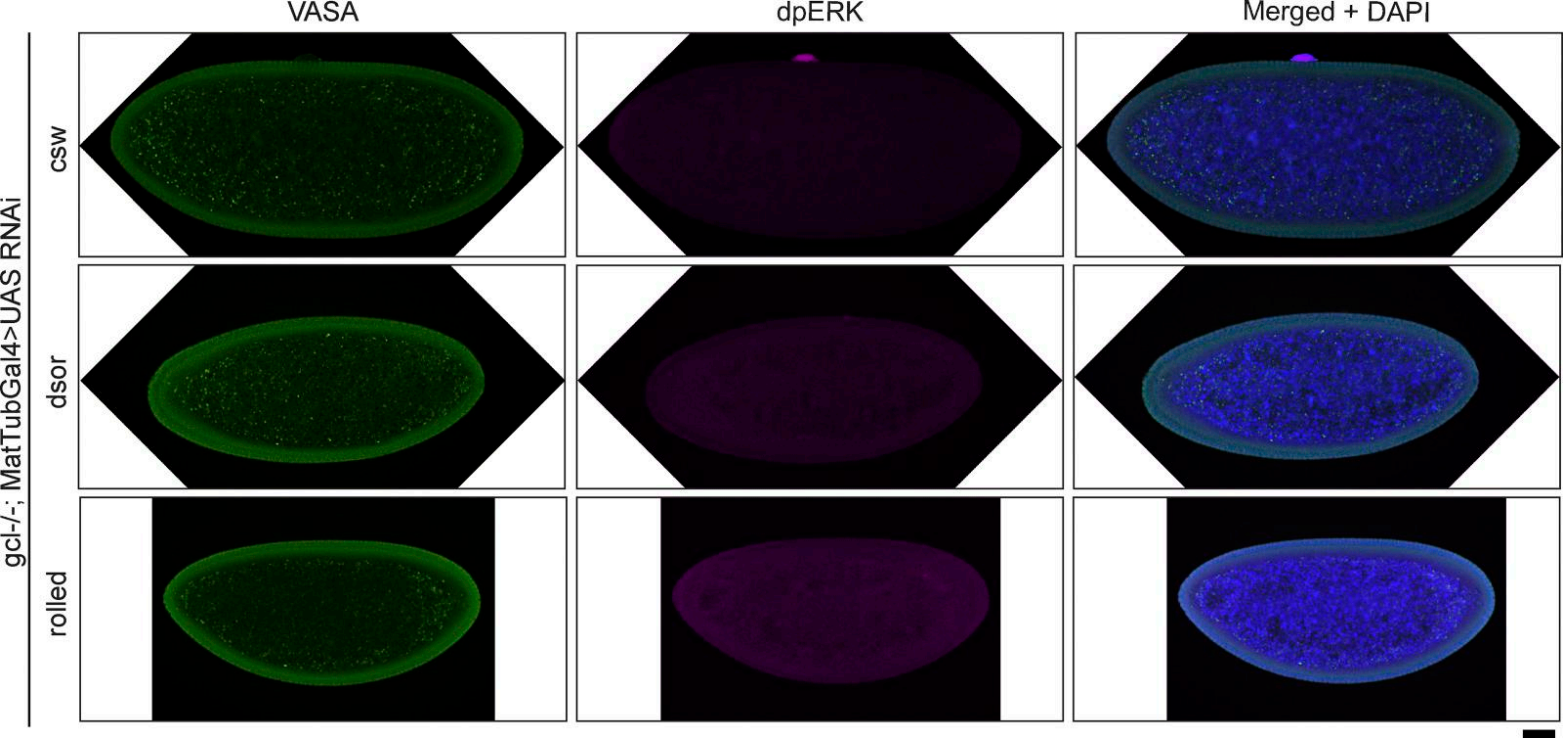
**(related to Fig. 2): Knockdown of *csw*, *dsor*, and *rolled* fails to rescue PGC defect in embryos from *gcl−/− mothers*.** Representative embryos of indicated maternal genotypes, nuclear cycle 13–14. Fixed embryos were immunostained with anti-Vasa (Green) and anti-dpERK (magenta). DNA (blue). Scale bar = 50 μm.

Since the optogenetic induction of Ras was sufficient to block PGC formation, we next examined which effectors downstream of Ras were necessary for this process. Since genetic disruption of Torso RTK restored PGC formation in *gcl−/−* embryos (Pae et al., 2017), we conducted RNAi against downstream components of the Torso pathway in *gcl−/−* mutant embryos and assessed whether PGC formation was similarly restored (Fig. 2, A–C and Fig. S1). As expected, based on genetic and optogenetic experiments, RNAi knockdown of *torso*, *shc*, *sos*, and *ras* restored PGC formation in *gcl−/−* embryos (Fig. 2 B). However, knockdown of *csw* (the fly homolog of SHP2), *raf*, *ksr*, *dsor1* (the fly homolog of MEK), and *rolled* (the fly homolog of MAPK) did not restore PGC formation (Pae et al., 2017) (Fig. S1). Csw (SHP2) has been previously shown to be a direct effector of RTKs. Our data suggest that, in the context of PGC formation, Csw may act in parallel or downstream of Ras and Raf signaling, consistent with similar findings for the sevenless RTK pathway in *Drosophila* photoreceptor development (Allard et al., 1996). To verify that RNAi effectively disrupted Torso signaling under these conditions, we stained for dpERK, a marker for MAPK phosphorylation, the final kinase step in canonical RTK signaling (Fig. 2 C and Fig. S1). All RNAi conditions except for mCherry RNAi displayed a loss of dpERK staining at the anterior and posterior poles. These results demonstrate that activation of the canonical RTK pathway downstream of Ras, including the Raf/MEK/MAPK signaling cascade, which plays a critical role in the transcriptional activation of somatic target genes, is neither necessary nor sufficient to interfere with PGC formation. We conclude that another, previously unknown effector of Torso RTK is involved in PGC formation.

### Ras separation-of-function mutants suggest that PI3K or RalA activation interferes with PGC pathway activation

**Figure 3.**
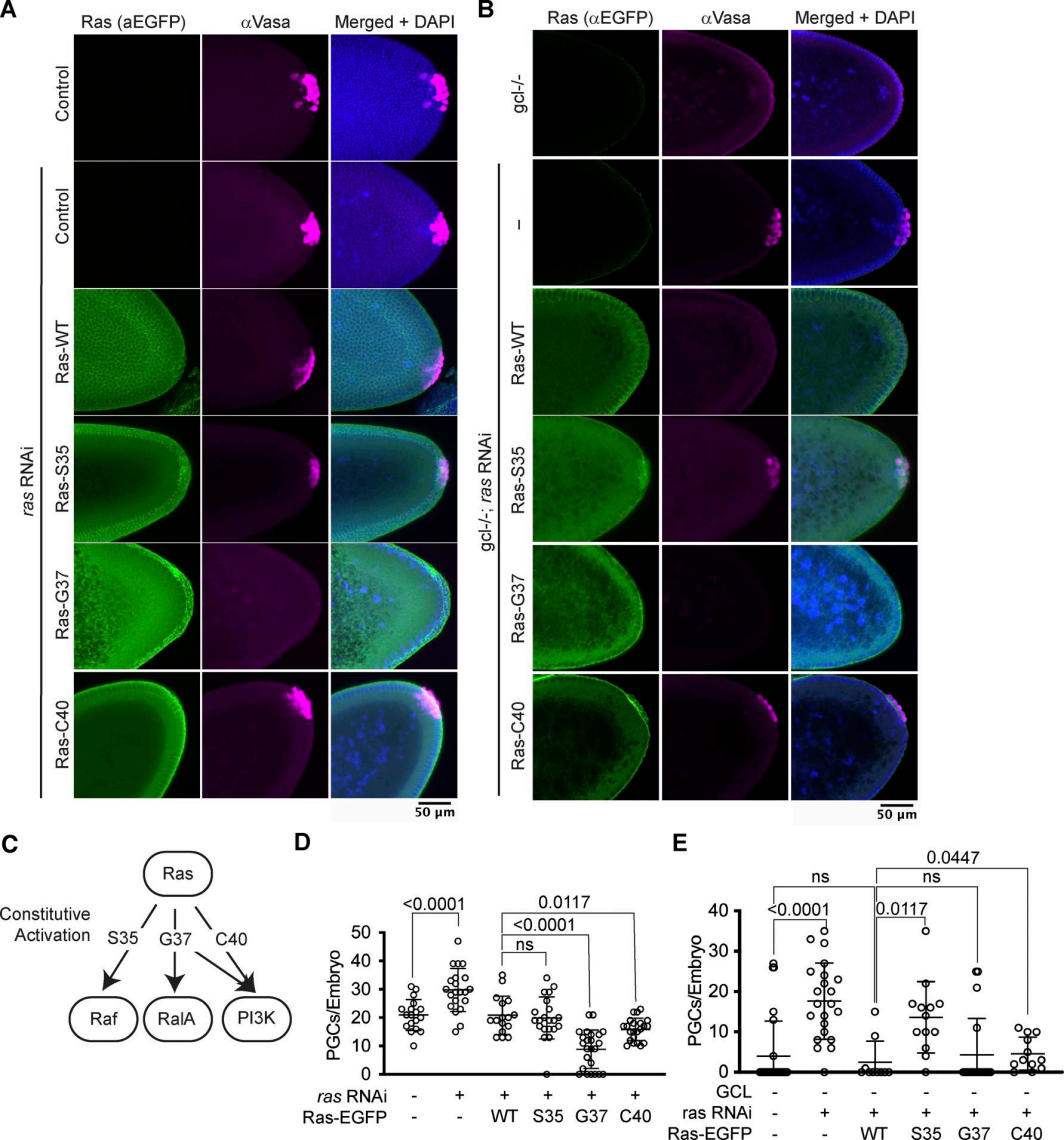
**Ras effector loop mutations that activate PI3K significantly decrease PGC formation, while Raf-activating** **mutations** **have no effect on PGC formation. (A)** Embryos derived from mothers depleted of endogenous Ras by Ras RNAi and expressing RNAi-insensitive EGFP-tagged Ras, Ras-S35, Ras-G37, and Ras-C40 were immunostained with anti-EGFP and anti-Vasa to confirm expression of the construct and stain PGCs for counting. Expression of the UASp constructs was driven in females by two copies of maternal-tubulin-GAL4-VP16 for optimal expression during late oogenesis. Images depict maximum intensity projections spanning area of PGC formation. **(B)** Embryos from *gcl−/−* mothers co-expressing Ras RNAi and RNAi-insensitive EGFP-tagged Ras, Ras-S35, Ras-G37, and Ras-C40 were immunostained with anti-EGFP and anti-Vasa to confirm expression of the construct and stain PGCs for counting. Expression of the UASp constructs was driven by maternal-tubulin-GAL4-VP16 for optimal expression during late oogenesis. Images depict maximum-intensity projections spanning the area of PGC formation. **(C)** Model depicting downstream pathways constitutively activated by Ras effector loop mutations. **(D)** PGCs in nuclear cycle 13–14 embryos from mothers of the indicated genotype were counted and plotted from A. The maternal genotype of control embryos is *w*^*1118*^. Bars represent the mean ± standard deviation. (*n* > 15, Mann–Whitney test). **(E)** PGCs in nuclear cycle 13–14 embryos from mothers of the indicated genotype were counted and plotted from B. The maternal genotype of control embryos is *w*^*1118*^. Bars represent the mean ± standard deviation. (*n* > 10, Mann–Whitney test).

To identify the signaling pathway downstream of Ras that may affect PGC formation, we focused on two additional, known downstream effectors of Ras: RalA and PI3K. The functions of these signaling pathways can be analyzed independently of the canonical MAP kinase pathway using a separation-of-function strategy. Point mutations in the effector loop domain of Ras act as molecular switches that, in addition to the Ras-V12 mutation, allow for the constitutive activation of a specific downstream pathway, while blocking Ras’s ability to activate other downstream pathways (Rodriguez-Viciana et al., 1997; Prober and Edgar, 2002). For example, the Ras-S35 mutation enables the constitutive activation of the Raf pathway while inhibiting Ras’s ability to activate RalA and PI3K (Fig. 3 C). To specifically separate Ras’s ability to activate its effectors, we knocked down endogenous *ras* expression with RNAi and expressed the effector loop mutants from RNAi-insensitive constructs (Fig. 3 A). Interestingly, knockdown of *ras* alone increased the number of PGCs by 30% compared with control embryos, demonstrating that Ras signaling counteracts PGC formation even in WT embryos, when GCL is present (Fig. 3 D).

Overexpression of WT Ras restored the number of PGCs to control levels. Embryos overexpressing Ras-S35 did not alter the number of PGCs formed, while embryos expressing Ras-G37 and Ras-C40 showed a reduction in PGCs, with a 57.5% reduction in Ras-G37 embryos (Fig. 3 D). Since Ras-G37 is known to activate both the RalA and the PI3K pathways, and Ras-C40 activates the PI3K pathway, our results suggest that these alternative pathways downstream of Ras interfere with PGC formation (Pacold et al., 2000; Kinashi et al., 2000).

Since Ras effector mutants are primarily thought to activate only one branch of the signaling pathway while inhibiting the other pathways, we used separation-of-function mutants to test which Ras effectors are targeted by GCL function. We expressed the Ras effector loop mutants in *gcl−/−* embryos alongside *ras* RNAi and measured PGC formation (Fig. 3 B). The expression of Ras-S35 rescued PGC formation in *gcl−/−* embryos, further demonstrating that the Ras–Raf pathway does not cause the PGC defect. The Ras-S35 mutant can no longer activate other downstream pathways of Ras, such as RalA and PI3K, thus blocking Ras’ ability to antagonize PGC formation. Overexpression of Ras-G37 could not rescue PGC formation in *gcl−/−* embryos, while overexpression of Ras-C40 was able to partially rescue PGC formation, though not to the same degree as Ras-S35 (Fig. 3 E). These results support the hypothesis that the activation of RalA and PI3K pathways antagonizes PGC formation and that this activation contributes to the PGC formation defects in *gcl−/−* embryos.

### PI3K activation is a major antagonist of PGC formation

Ras separation-of-function mutants identified two potential pathways downstream of Ras whose activation may affect PGC formation: RalA, a GTPase that recruits the exocyst complex and plays a role in membrane addition during somatic cellularization (Holly et al., 2015), and PI3K, a key regulator of metabolism and cellular morphogenesis which phosphorylates phosphatidylinositol 4,5-bisphosphate (PIP2) to generate phosphatidylinositol 3,4,5-trisphosphate (PIP3) (Stambolic et al., 1998; Shewan et al., 2011). To directly determine whether the activity of these effectors can interfere with PGC formation specifically, we expressed variants of RalA and PI3K subunits at the posterior pole using a germplasm-specific 3′UTR containing the translational control element of *nanos* and the 3′UTR of *pgc* (TCEp3) (Lin et al., 2020).

**Figure S2.**
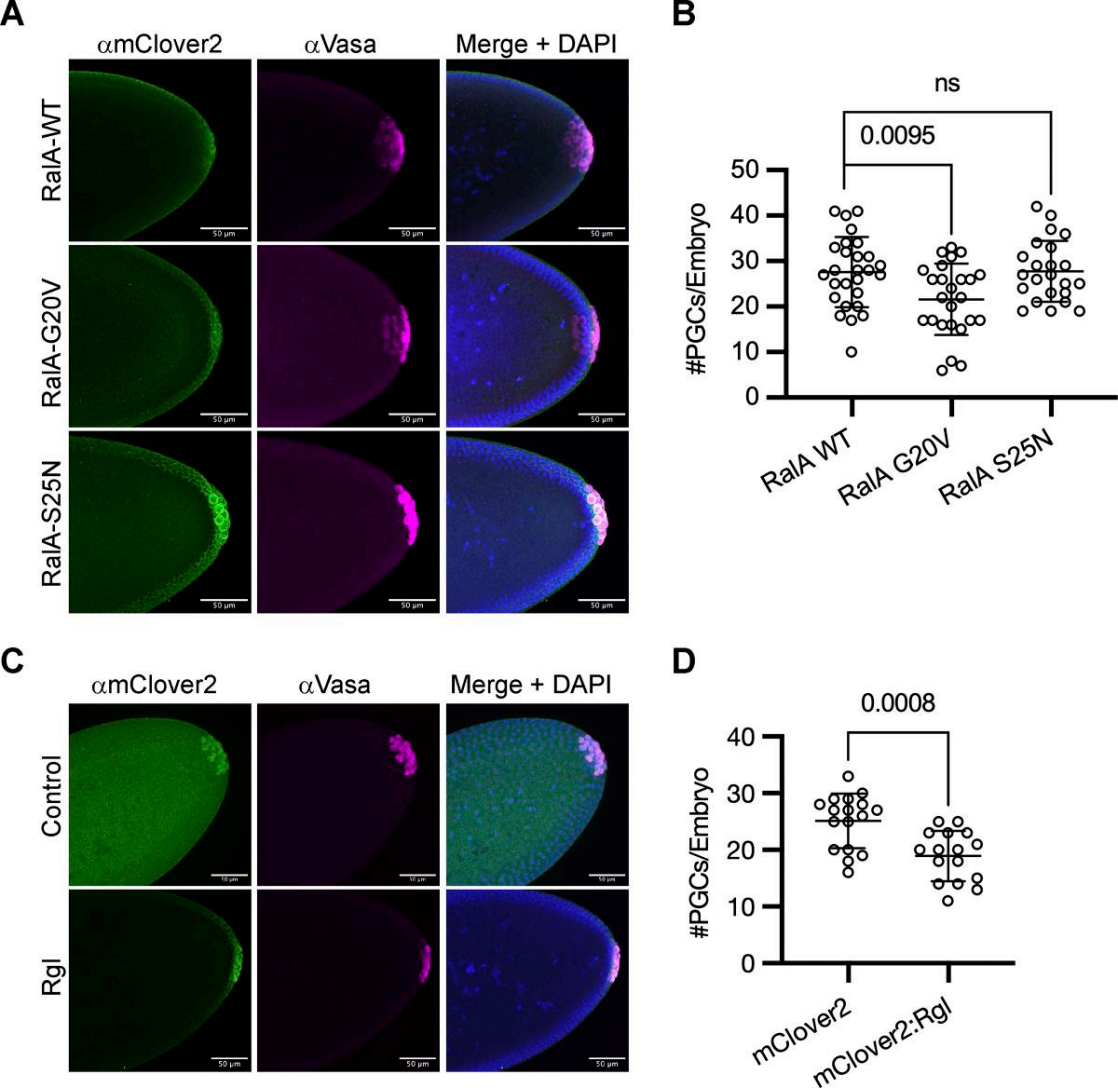
**(related to Fig. 3): Increasing RalA activity mildly antagonizes PGC formation. (A)** Embryos from mothers overexpressing mClover2-tagged RalA-WT, RalA-G20V (constitutively active), and RalA-S25N (dominant negative) were immunostained with anti-mClover2 and anti-Vasa to confirm expression of the construct and stain PGCs for counting. Expression of the respective UASp constructs was driven by two copies of maternal-tubulin-GAL4-VP16 for optimal expression during late oogenesis. Images depict maximum-intensity projections spanning the region of PGC formation. Scale bar = 50 µm. **(B)** PGCs in nuclear cycle 13–14 embryos from mothers of the indicated genotype were counted and plotted from A. Overexpression of RalA-WT was used as a control. Bars represent the mean ± standard deviation. (*n* > 20, Mann–Whitney test). **(C)** Embryos from mothers overexpressing mClover2-tagged Rgl were immunostained with anti-mClover2 and anti-Vasa to confirm expression of the construct and stain PGCs for counting. Expression of the UASp construct was driven by two copies of maternal-tubulin-GAL4-VP16 for optimal expression during late oogenesis. Images depict maximum intensity projections spanning the area of PGC formation. Scale bar = 50 µm. **(D)** The number of PGCs in nuclear cycle 13–14 embryos from mothers of the indicated genotype was counted and plotted from B. Overexpression of germline-targeted mClover2 was used as a control. Bars represent the mean ± standard deviation. (*n* > 15, Mann–Whitney test).

**Figure 4.**
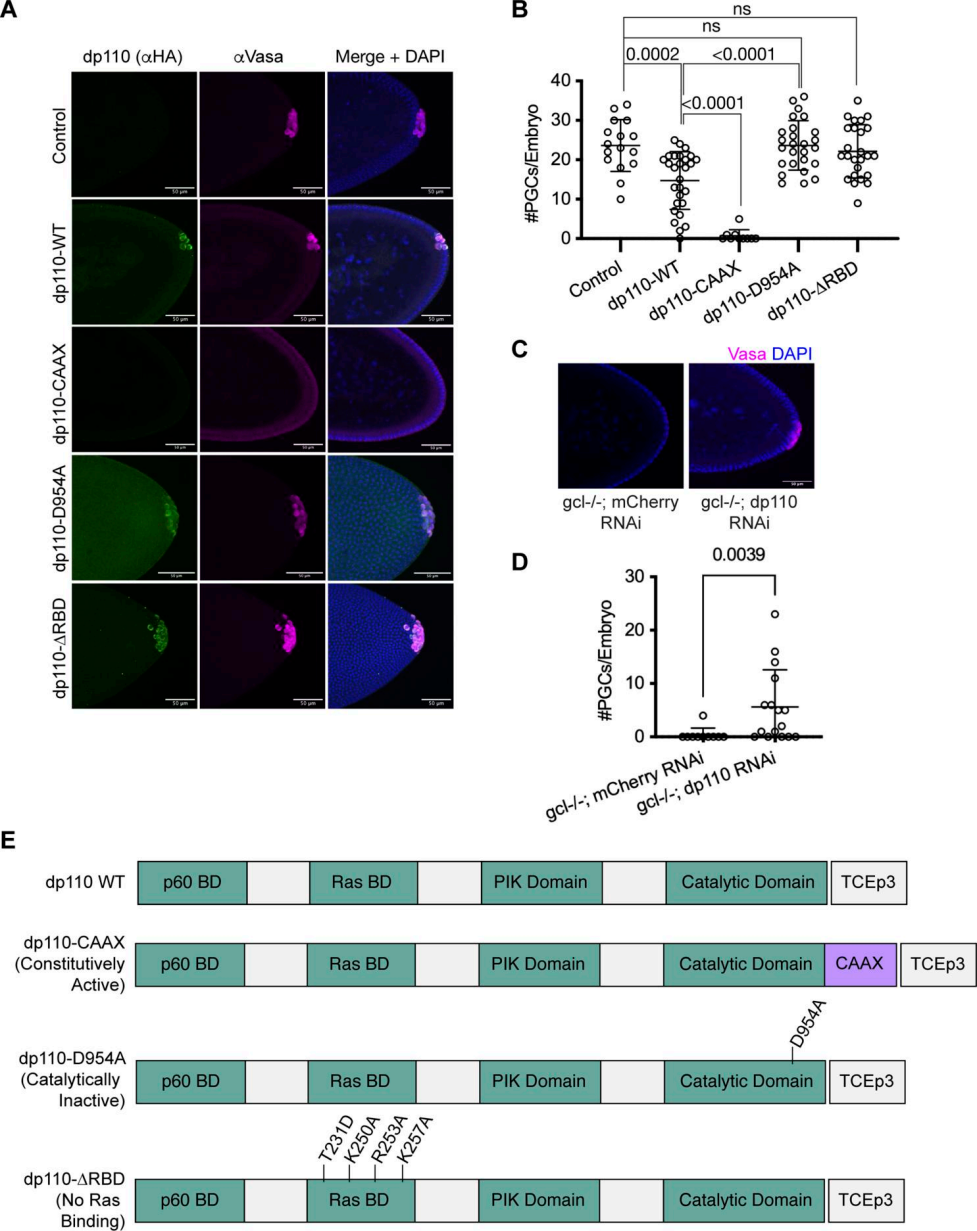
**PI3K strongly antagonizes PGC formation through its Ras-binding and catalytic activity. (A)** Embryos from mothers overexpressing germplasm-targeted, HA-tagged dp110-WT, dp110-CAAX (constitutively active), dp110-D954A (catalytically inactive), and dp110-ΔRBD (no Ras binding) were immunostained with anti-HA and anti-Vasa to confirm expression of the construct and stain PGCs for counting. Maternal expression of the UASp constructs was driven by two copies of maternal-tubulin-GAL4-VP16 for optimal expression during late oogenesis. Images depict maximum intensity projections spanning the area of PGC formation. Scale bar = 50 µm. **(B)** PGCs in nuclear cycle 13–14 embryos from mothers of the indicated genotype were counted and plotted from A. The genotype of control mothers is maternal-tubulin-GAL4-VP16/+; maternal-tubulin-GAL4-VP16/+. Embryos from control, dp110-D954A, and dp110-ΔRBD mothers show no significant difference in PGC amounts. Bars represent the mean ± standard deviation. (*n* > 10, Mann–Whitney test). **(C)** Embryos from *gcl−/−* mothers expressing RNAi against dp110 were immunostained with anti-Vasa to count PGCs. Images depict maximum intensity projections spanning the area of PGC formation. Scale bar = 50 µm. **(D)** PGCs in nuclear cycle 13–14 embryos from mothers of the indicated genotype were counted and plotted from C. RNAi against mCherry was used as a control. Bars represent the mean ± standard deviation. (*n* > 10, Mann–Whitney test). **(E)** Schematic depicting dp110-WT and variant constructs used. All constructs contain the germplasm-targeting 3′UTR (TCEp3).

We generated constitutively active and dominant-negative mutations in RalA: RalA-G20V and RalA-S25N, respectively. For human RalA, these mutations have been shown to modulate RalA activity by changing its binding affinity to GDP and GTP (Hinoi et al., 1996; Sawamoto et al., 1999). Germplasm-specific overexpression of RalA-G20V slightly decreased PGC formation by 21.7%, while germplasm overexpression of RalA-S25N, a dominant-negative allele of RalA, had no effect (Fig. S2, A and B). Similarly, germplasm overexpression of the RalGEF Rgl, which is expected to increase RalA activity, only slightly decreased PGC formation by 24.6% (Fig. S2, C and D). In contrast, increasing PI3K activity had a robust antagonistic effect on PGC formation. PI3K activity relies on a catalytic subunit, p110 in mammals and dp110 in *Drosophila*, as well as a regulatory subunit, p85 in mammals and p60 in *Drosophila*. Germplasm overexpression of the WT catalytic domain dp110 significantly reduced PGC formation by 37.6% (Fig. 4, A and B). Further increasing PI3K activity by overexpressing membrane-targeted dp110-CAAX had an even stronger effect, reducing PGC formation by 97.0% (Fig. 4, A and B). While our results suggest that activation of RalA and PI3K can counteract PGC formation, we observed more robust effects by manipulating the PI3K pathway. Indeed, while Ras-C40 and Ras-G37 have both been shown to activate PI3K signaling, it has previously been reported that expression of Ras-G37 but not Ras-C40 or Ras-S35 results in increased PIP3 levels in *Drosophila* wing tissues (Prober and Edgar, 2002). We therefore focused our subsequent analysis on the mechanisms by which the PI3K pathway is regulated at the posterior pole and how this regulation affects PGC formation.

To test whether dp110’s catalytic activity was required for its suppression of PGC formation, we overexpressed germplasm-targeted (TCEp3) dp110-D954A, which can no longer bind to ATP and has been shown to have a dominant-negative phenotype when overexpressed (Fig. 4 E) (Leevers et al., 1996). These embryos formed the same amount of PGCs as WT control, suggesting that ATP-binding and catalytic activity are required for dp110 to suppress PGC formation (Fig. 4, A and B). To test whether the binding of the PI3K catalytic subunit to Ras mediated the effect on PGC formation, we mutated the Ras-binding domain (RBD) of dp110. Four-point mutations in the RBD have been shown to block dp110’s ability to bind to Ras and decrease the maximal signaling of PI3K, even though the catalytic domain remains intact (Fig. 4 E) (Orme et al., 2006). Consistent with a direct interaction between Ras and PI3K, dp110-ΔRBD overexpression did not affect PGC formation (Fig. 4, A and B).

Next, we inquired whether inappropriate PI3K activation could explain the defects observed in *gcl−/−* embryos. If so, we would expect that decreasing dp110 expression would restore PGC formation in *gcl−/−* embryos. Indeed, RNAi knockdown of the PI3K dp110 subunit in *gcl−/−* embryos partially rescued PGC formation, with 50% of embryos making five or more PGCs compared with 0% in control (Fig. 4, C and D). These experiments identify PI3K as a direct mediator of Torso activation in antagonizing PGC formation.

### Suppression of PI3K activity results in ectopic cellularization only at the posterior pole

**Figure S3.**
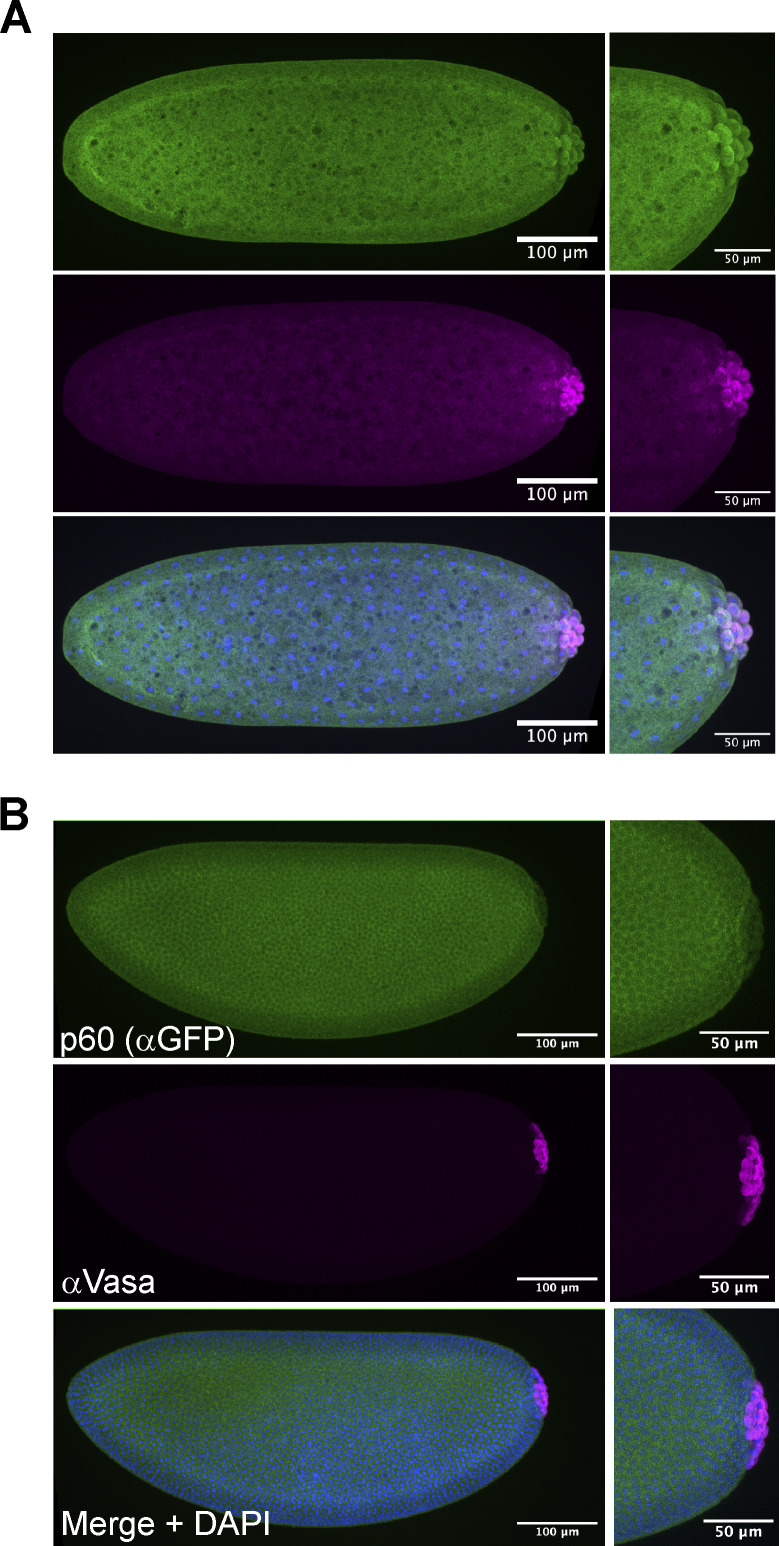
**(related to Fig. 5): p60, the regulatory subunit of PI3K in *Drosophila*, is uniformly distributed across the embryo prior to PGC formation, but by nuclear cycle 14 localizes to furrows and is excluded from pole cells. (A)** Nuclear cycle 9–10 embryos shortly before pole cell formation from mothers expressing a p60-GFP fosmid were immunostained for anti-GFP and anti-Vasa. The inset shows a close-up of the posterior pole. Images depict maximum intensity projections spanning the area of PGC formation. Scale bar = 100 µm for whole embryo, 50 µm for inset **(B)** Nuclear cycle 13–14 embryos after pole cell formation from mothers expressing a p60-GFP fosmid were immunostained for anti-GFP and anti-Vasa. The inset shows a close-up of the posterior pole. Images depict maximum intensity projections spanning the area of PGC formation. Scale bar = 100 µm for whole embryo, 50 µm for inset.

**Figure 5.**
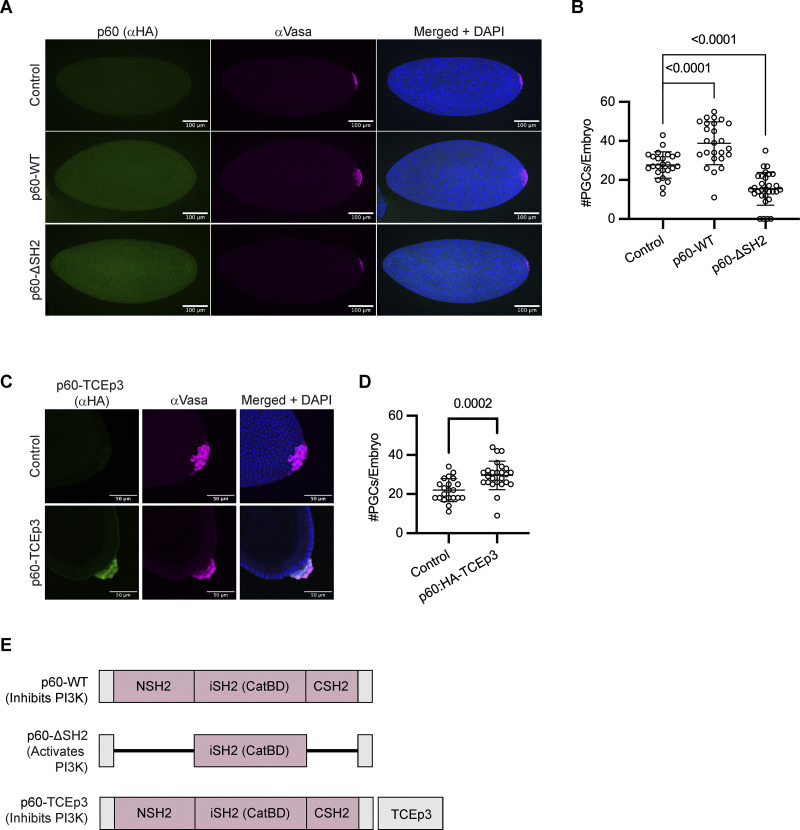
**Decreasing PI3K activity rescues the *gcl−/−* phenotype and increases PGC formation. (A)** Embryos from mothers overexpressing HA-tagged p60-WT and p60-ΔSH2 were immunostained with anti-HA and anti-Vasa to confirm expression and count PGCs. Images depict maximum intensity projections spanning the area of PGC formation. Scale bar = 100 µm. **(B)** PGCs in nuclear cycle 13–14 embryos from mothers of indicated genotype were counted and plotted from A. The maternal genotype of control embryos is maternal-tubulin-GAL4-VP16/+; maternal-tubulin-GAL4-VP16/+. Bars represent the mean ± standard deviation. (*n* > 20, Mann–Whitney test). **(C)** Embryos from mothers overexpressing HA-tagged p60 with a germplasm-targeting 3′UTR (TCEp3) were immunostained with anti-HA and anti-Vasa to confirm expression and count PGCs. Images depict maximum intensity projections spanning the area of PGC formation. Scale bar = 50 µm. **(D)** PGCs in nuclear cycle 13–14 embryos from mothers of the indicated genotype were counted and plotted from C. The maternal genotype of control embryos is maternal-tubulin-GAL4-VP16/+; maternal-tubulin-GAL4-VP16/+. Bars represent the mean ± standard deviation. (*n* > 20, Mann–Whitney test). **(E)** Schematic depicting p60-WT and variant constructs used.

To determine how PI3K activation may interfere with PGC formation, a cellular event involving membrane budding and localized membrane constriction, we focused on the role of PI3K in converting PIP2 to PIP3. This conversion relies on the regulatory subunit of PI3K, p60, which functions as a switch that mediates the plasma membrane localization of dp110 upon Ras activation (Leevers et al., 1996). The *pi3K21B* gene encodes the p60 subunit in *Drosophila*, and *pi3k21b* mRNA is enriched in the germplasm of early embryos and in PGCs after their formation (Xi et al., 2014). Imaging of a sfGFP-tagged fosmid line revealed that p60 protein is present in high levels throughout the early embryo (Fig. S3, A and B). In the absence of RTK activation, p60 binds to dp110 and inhibits PI3K function (Leevers et al., 1996). Therefore, increasing p60 expression may further protect pole buds from PI3K activity. To test this idea, we overexpressed p60 uniformly across the embryo (Tamada et al., 2021). Embryo-wide overexpression of p60 resulted in an increase in PGC formation compared with the control, demonstrating the ability of p60 to suppress PI3K activity during PGC formation (Fig. 5, A and B). Despite this, there was no evidence of ectopic cellularization of the soma during nuclear cycle 14 when p60 was overexpressed, indicating that this effect is specific to the posterior pole where germplasm is enriched. The SH2 domains of PI3K’s regulatory subunit inhibit PI3K activity, and removal of the SH2 domains has been shown to increase PI3K activity (Fig. 5 E) (Klippel et al., 1994). When we overexpressed p60-ΔSH2, we observed a decrease in PGC formation similar to membrane-targeted dp110, supporting the conclusion that p60 can act as a modulator of PI3K signaling that is particularly critical for PGC formation (Fig. 5, A and B).

Overexpression of WT p60 using the TCEp3 germplasm-targeting 3′UTR (TCEp3) corroborated this result by increasing PGC formation compared with the control. In addition, PGCs appeared to protrude more from the embryo’s somatic blastoderm and packed more tightly on top of each other than in the control (Fig. 5 C). This contrasts with control embryos, where PGCs form a less compact monolayer that wraps around the posterior pole. Our results clearly demonstrate a connection between PI3K activity and PGC formation: low levels of PI3K activity support PGC formation, whereas high levels antagonize it.

### Spatially restricted Torso activity patterns PIP3 in the embryo

**Figure 6.**
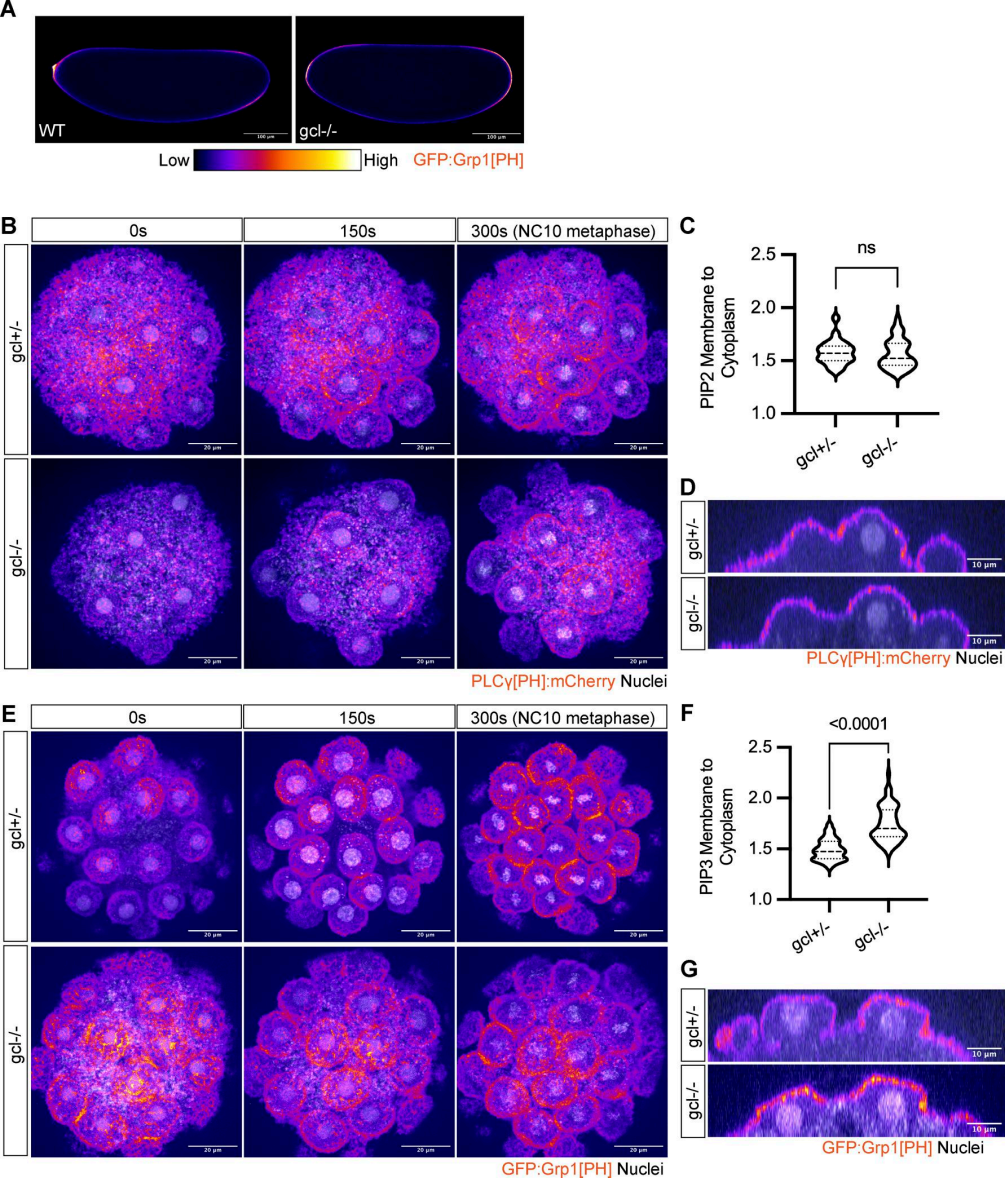
**PIP3 acts downstream of Torso and is inappropriately enriched in the posterior pole membrane when GCL is absent. (A)** Embryos from WT and *gcl−/−* mothers also expressing the PIP3 biosensor GFP:Grp1[PH] (fire LUT) were live imaged on their side to visualize PIP3 spatial distribution. Embryos are less than an hour old, since nuclei have not yet migrated to the cortex. Scale bar = 100 µm. Lateral view of embryos oriented with their anterior poles to the left and their posterior poles to the right. Images show a single plane roughly through the middle of the embryo. Scale bar = 100 µm. **(B)** Embryos from *g*+*/−* and g*cl−/−* mothers at embryonic nuclear cycle 10 expressing the PIP2 biosensor PLCγ[PH]:mCherry (fire LUT) and nuclear marker His2AV:GFP (gray) were posteriorly mounted and live imaged. Images are maximum intensity projections of a 20 µm section. The 300-s time point represents nuclear cycle 10 metaphase. Scale bar = 20 µm. **(C)** Membrane and cytoplasm were segmented from posterior-mounted embryo movies 150 s before nuclear cycle 10 metaphase, and the membrane-to-cytoplasm ratio of PIP2 fluorescence was plotted. (*n* = 6 embryos for *g*+*/−* maternal genotype, *n* = 8 embryos for *gcl−/−* maternal genotype, Mann–Whitney test). **(D)** Orthogonal view of embryos from *g*+*/−* and *gcl−/−* mothers expressing the PIP2 biosensor PLCγ[PH]:mCherry (fire LUT) and nuclear marker His2AV:GFP (gray) 150 s before nuclear cycle 10 metaphase. Scale bar = 10 µm. **(E)** Embryos from *g*+*/−* and *gcl−/−* mutant mothers also maternally expressing the PIP3 biosensor GFP:Grp1[PH] (fire LUT) and nuclear marker His2AV:RFP (gray) were posteriorly mounted and live imaged at embryonic nuclear cycle 10. Images are maximum intensity projections of a 20 µm section. The 300-s time point represents nuclear cycle 10 metaphase. Scale bar = 20 µm. **(F)** Membrane and cytoplasm were segmented from posterior-mounted embryo movies 150 s before nuclear cycle 10 metaphase, and the membrane-to-cytoplasm ratio of PIP3 fluorescence was plotted. (*n* = 6 embryos for *g*+*/−* maternal genotype, *n* = 5 embryos for *gcl−/−* maternal genotype, Mann–Whitney test). **(G)** Orthogonal view of embryos from *g*+*/−* and *gcl−/−* mothers also expressing the PIP3 biosensor GFP:Grp1[PH] (fire LUT) and nuclear marker His2AV:RFP (gray) imaged 150 s before nuclear cycle 10 metaphase. Scale bar = 10 µm.

PI3K catalyzes the production of PIP3 by phosphorylating PIP2. To understand how this production is impacted by GCL-mediated degradation of Torso at the posterior pole, we analyzed the distribution of PIP3 in WT and *gcl−/−* embryos using the biosensor GFP:Grp1[PH] (Prober and Edgar, 2002). In early WT embryos, before nuclei have reached the embryo cortex, PIP3 was enriched at the membrane in areas of high Torso activity: the anterior pole and the regions adjacent to the posterior pole but was excluded from the posterior pole where germplasm is localized (Fig. 6 A). In *gcl−/−* embryos, PIP3 was no longer excluded from the posterior pole. Thus, by promoting degradation of the Torso RTK, GCL prevents PIP3 generation specifically at the posterior pole where it is translated.

**Figure S4.**
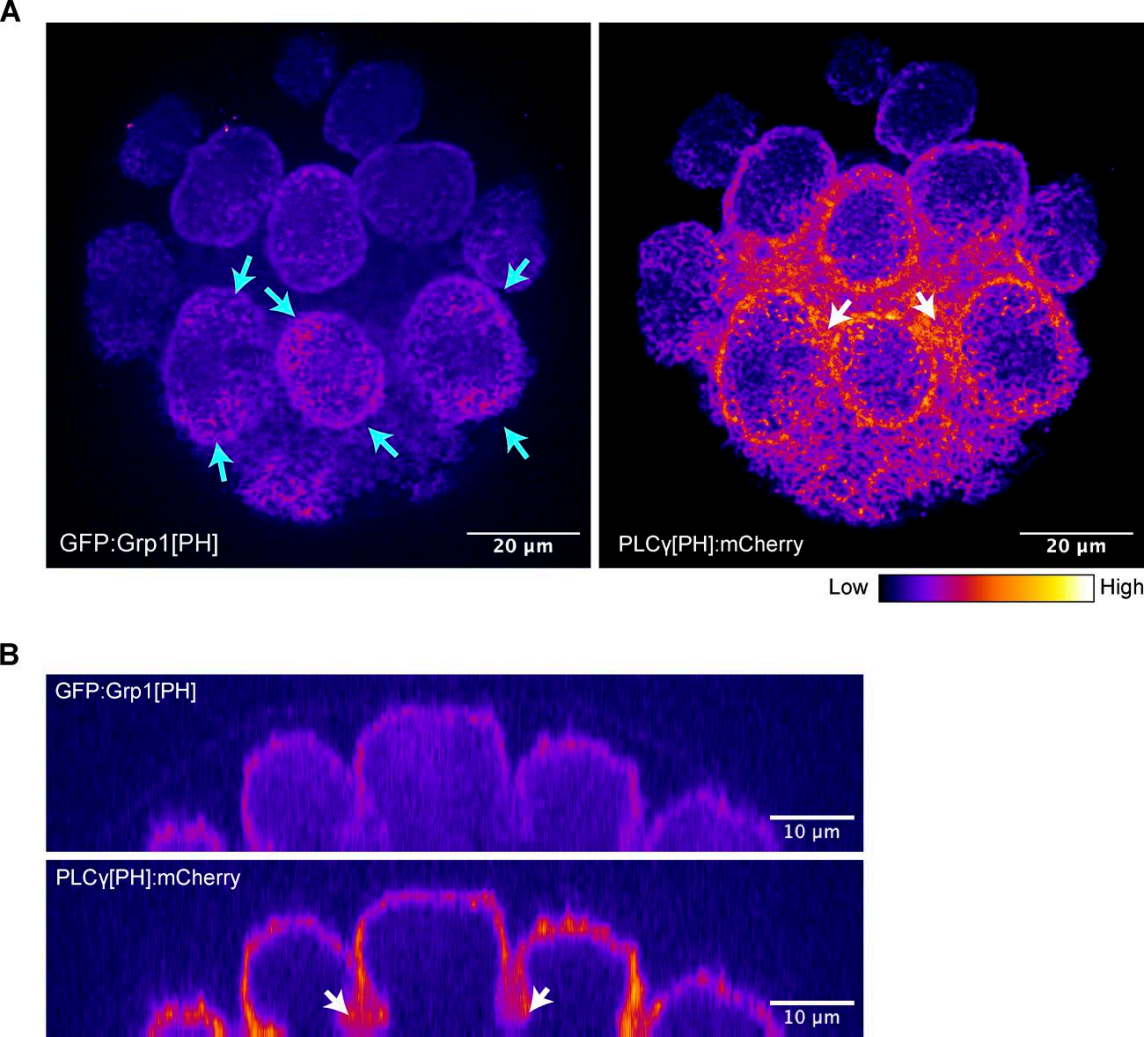
**(related to Fig. 7): PIP2 and PIP3 occupy separate membrane compartments of the embryo and the posterior pole. (A)** Embryos from mothers expressing GFP:Grp1[PH] and PLCγ[PH]:mCherry were live imaged, mounted on their posterior pole. The embryo is in nuclear cycle 10, prior to PGC formation. Timing is approximately 5 min before mitosis. Enrichment of the PIP3 biosensor is seen at the poles of buds (cyan arrows). PIP2 is enriched in the interbud regions (white arrows). The image shows the maximum intensity projection of a 20 µm section. Images are pseudo-colored with the fire LUT. Scale bar = 20 µm. **(B)** Orthogonal view of the embryo from B to visualize pole bud furrows. PIP2 but not PIP3 is enriched at the bud furrow (white arrows). Images are pseudo-colored with the fire LUT. Scale bar = 20 µm.

PIP2 has recently been shown to be enriched at the posterior pole in the cellularizing pole buds (Kilwein et al., 2025). We used the PIP2 biosensor PLCγ[PH]:mCherry and the PIP3 biosensor GFP:Grp1[PH] to simultaneously observe the distribution of these phosphoinositide species in developing embryos. While PIP3 is restricted to membrane regions with high Torso activity, PIP2 exhibits a more dynamic localization, enriching at either the anterior or posterior pole depending on the movement of the membrane during syncytial nuclear divisions (Video 1). Examining the dynamic localization of these phosphoinositides in pole buds by live imaging, we observed that PIP3 is depleted from the base of pole buds, while PIP2 is enriched at the base and throughout the bud membrane (Fig. S4, A and B). To measure if there was a change in PIP2 and PIP3 levels in the membrane of *gcl−/−* pole buds, we live-imaged either PLCγ[PH]:mCherry or GFP:Grp1[PH] in posteriorly mounted embryos along with the nuclear marker His2AV:GFP or His2AV:RFP. This enabled precise embryo staging and pole bud segmentation, thereby capturing membrane dynamics during nuclear cycle 10 immediately preceding PGC formation. The membrane enrichment of PIP3, as measured by the membrane-to-cytoplasm ratio of PIP3 fluorescence intensity, is higher in *gcl−/−* nuclear cycle 10 posterior pole buds compared with *gcl*+/− pole buds, while there is no difference in PIP2 fluorescence intensity (Fig. 6, B–G). This suggests that the previously reported posterior enrichment of PIP2 is independent of GCL’s function (Kilwein et al., 2025). This also aligns with previous observations that the PI3K pathway does not significantly affect PIP2 levels (Jackson et al., 1992).

**Figure 7.**
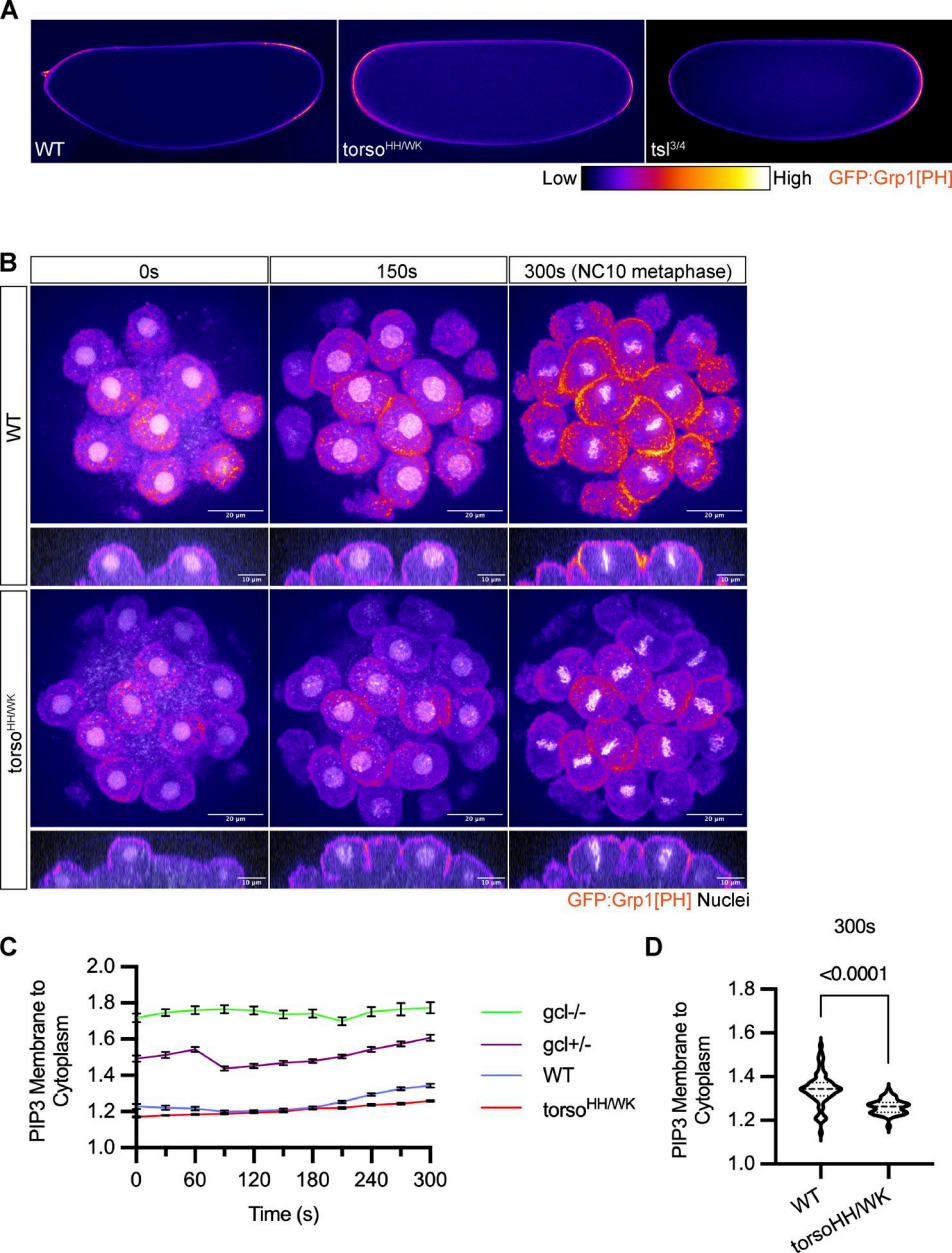
**PIP3 levels in the embryo are dependent on Torso dosage. (A)** Embryos from WT, *torso*^*HH/WK*^, and *tsl*^*3/4*^ mothers also expressing the PIP3 biosensor GFP:Grp1[PH] (fire LUT) were live-imaged to visualize PIP3 spatial distribution. Embryos are imaged prior to nuclear cycle 9, since the nuclei have not yet migrated to the cortex. Embryos are oriented with their anterior poles to the left and their posterior poles to the right. Images show a single plane roughly through the middle of the embryo. **(B)** Embryos from WT and *torso*^*HH/WK*^ mutant mothers also expressing the PIP3 biosensor GFP:Grp1[PH] (fire LUT) and nuclear marker His2AV:RFP (gray) were posteriorly mounted and live imaged at nuclear cycle 10. Images are maximum intensity projections of a 20 µm section. The 300-s time point represents nuclear cycle 10 metaphase. Scale bar = 20 µm. Orthogonal views are shown below maximum intensity projections. Scale bar = 10 µm. **(C)** Time-lapse measurement of PIP3 membrane-to-cytoplasm ratio in embryos from WT, *gcl*+/−, *gcl−/−*, and *torso*^*HH/WK*^ mothers. Bars represent the mean ± SEM. **(D)** The membrane-to-cytoplasm ratio of PIP3 fluorescence was plotted for embryos from WT and *torso*^*HH/WK*^ mutant mothers at nuclear cycle 10 metaphase (300 s timepoint). (*n* = 7 embryos from WT mothers, *n* = 7 embryos from *torso*^*HH/WK*^ mothers, Mann–Whitney test).

**Video 1.** **(related to Fig. 7 and Fig. S4): PIP2 and PIP3 membrane compartments in a laterally mounted embryo.** A representative embryo from a mother expressing the PIP3 biosensor GFP:Grp1[PH] and the PIP2 biosensor PLCγ[PH]:mCherry. Embryos were live-imaged every thirty seconds, mounted laterally. The movie starts when the embryo is in nuclear cycle 10, prior to PGC formation. The image shows a single plane roughly in the middle of the embryo. In the Merge panel, GFP: Grp1[PH] is pseudo-colored in green and PLCγ[PH]:mCherry is pseudo-colored in magenta. Scale bar = 50 µm.

To test whether loss of Torso signaling abolishes the spatial patterning of PIP3, we imaged GFP:Grp1[PH] in embryos from loss-of-function *torso*^*HH/WK*^ mutant mothers (Fig. 7, A and B). While we no longer observed a WT pattern of PIP3, the biosensor appeared to expand to the anterior and posterior poles in early embryos before nuclei reach the cortex. We tested whether Torso-like, which can have roles independent of Torso RTK signaling, may contribute to the terminally expanded PIP3 patterning (Taylor et al., 2019). We imaged GFP:Grp1[PH] in embryos from *tsl*^*3/4*^ mothers and observed that the terminal enrichment of PIP3 persisted as in *torso* mutant embryos (Fig. 7 A). We hypothesize that in the absence of PIP3 production, the strongly expressed biosensor may bind promiscuously to other phosphoinositides, since the PH domain of Grp1 has been shown to have some nonspecific binding (Li et al., 2020). To more directly determine the sensitivity of PIP3 levels to Torso activity at the time of PGC formation, we compared PIP3 membrane enrichment in embryos with various levels of posterior pole Torso activity. As expected for GCL-dependent degradation of Torso, the membrane-to-cytoplasm ratio of GFP:Grp1[PH] was indistinguishable between WT and *torso*^*HH/WK*^ pole buds at 150 s before nuclear cycle 10 (Fig. 7, B and C). Lower levels of PIP3 were observed at the onset of metaphase at the 300-s time point in *torso*^*HH/WK*^ pole buds, indicating that WT posterior pole buds have latent levels of Torso and PI3K signaling that escape GCL-mediated degradation (Fig. 7 D). PIP3 levels were significantly elevated in *gcl−/−* pole buds compared with WT. Interestingly, we also observed higher PIP3 levels in *gcl*+*/−* pole buds, demonstrating the sensitivity of PIP3 to Torso activity (Fig. 7 C). This also suggests there is a threshold level of PIP3 that can be tolerated for PGC formation, as *gcl*+*/−* embryos produce approximately the same number of PGCs as WT.

Together, these results suggest that the spatially restricted activation of Torso at the egg termini patterns PI3K activity and PIP3 enrichment in the early embryo. GCL-dependent degradation of Torso at the posterior pole causes asymmetrical membrane remodeling at the site of PGC formation.

### Pole buds in *gcl−/−* embryos show defective myosin assembly

**Figure 8.**
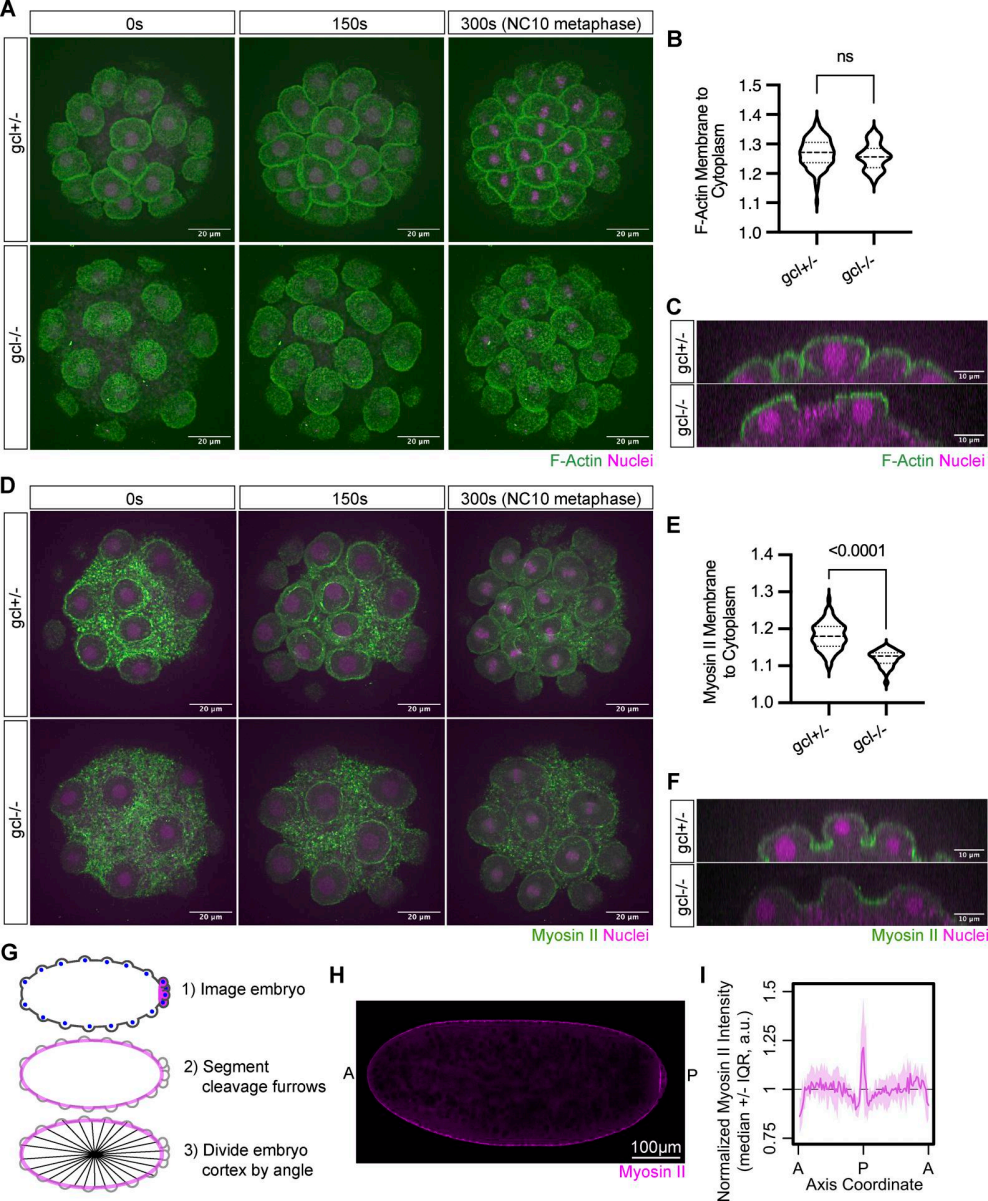
**Torso/PI3K activity decreases myosin II recruitment to the pole bud membrane. (A)** Embryos from *gcl*+*/−* and *gcl−/−* mothers also expressing the F-actin marker moe[ABD]:GFP (green) and the nuclear marker His2AV:RFP (magenta) were posteriorly mounted and live imaged. Images are maximum intensity projections of a 20 µm section. The 300 s time point represents nuclear cycle 10 metaphase. Scale bar = 20 µm. **(B)** Membrane and cytoplasm were segmented from posterior-mounted embryo movies 150 s before nuclear cycle 10 metaphase, and the membrane-to-cytoplasm ratio of F-Actin fluorescence was plotted. (*n* = 6 embryos for *gcl*+*/−* maternal genotype, *n* = 5 embryos for *gcl−/−* maternal genotype, Mann–Whitney test). **(C)** Orthogonal view of embryos from *gcl*+*/−* and *gcl−/−* mothers also expressing the F-actin marker moe[ABD]:GFP (green) and the nuclear marker His2AV:RFP (magenta) 150 s before nuclear cycle 10 metaphase. Scale bar = 10 µm. **(D)** Embryos from *gcl*+*/−* and *gcl−/−* mothers also expressing a marker for the regulatory light chain of non-muscle myosin II, sqh:3xGFP (green) and the nuclear marker His2AV:RFP (magenta) were posteriorly mounted and live imaged. Images are maximum intensity projections of a 20 µm section. The 300-s time point represents nuclear cycle 10 metaphase. Scale bar = 20 µm. **(E)** Membrane and cytoplasm were segmented from posterior-mounted embryo movies 150s before nuclear cycle 10 metaphase, and the membrane-to-cytoplasm ratio of myosin II fluorescence was plotted. (*n* = 6 embryos for *gcl*+*/−* maternal genotype, *n* = 5 embryos for *gcl−/−* maternal genotype, Mann–Whitney test). **(F)** Orthogonal view of embryos from *gcl*+*/−* and *gcl−/−* mothers also expressing a marker for the regulatory light chain of non-muscle II, sqh:3xGFP (green) and the nuclear marker His2AV:RFP (magenta) 150 s before nuclear cycle 10 metaphase. Scale bar = 10 µm. **(G)** Schematic of embryo segmentation for the quantification in I. **(H)** Nuclear cycle 11–12 embryos from mothers expressing a marker for the regulatory light chain of non-muscle myosin II, sqh:mScarlet-I (magenta), were fixed and laterally mounted. The embryo shown is oriented with its anterior (A) to the left and its posterior (P) to the right. The image shows a single plane roughly in the middle of the embryo. Scale bar = 100 µm. **(I)** Plot of myosin II fluorescence intensity across the circumference of the embryo cortex, normalized to the average fluorescence intensity of the cleavage furrows per embryo. *n* = 25 embryos.

Cellular morphogenesis events such as cytokinesis and cell migration depend on precise spatiotemporal regulation of PIP2 and PIP3 to effectively balance protrusive and contractile forces (Shewan et al., 2011). During *Drosophila* somatic cellularization, excessive PIP3 results in failed cellularization due to myosin disassembly, while excessive PIP2 causes premature cellularization due to increased contractility (Reversi et al., 2014). The architecture of the round, protrusive pole bud relies significantly on the counterforces between the cortical stiffness provided by the F-actin–rich cap and the contractility generated by the actomyosin-rich base (Fernandez-Gonzalez and Harris, 2023). Elevated PIP3 levels often indicate rapid actin polymerization, particularly at the leading edges of migrating cells (Devreotes and Janetopoulos, 2003). To investigate whether PIP3 levels correlated with F-actin polymerization in the caps of pole buds during PGC formation, we live-imaged the actin-binding domain of moesin fused to GFP (GFP:moe[ABD]). GFP:moe[ABD] serves as a reliable marker of F-actin that does not interfere with endogenous actin dynamics (Spracklen et al., 2014). Our findings revealed that in *gcl−/−* embryo nuclear cycle 10 pole buds, F-actin appeared disorganized and roughened, but the fluorescence intensity did not increase when compared with *gcl*+*/−* pole buds, suggesting that actin polymerization did not increase (Fig. 8, A–C). The roughened appearance of the F-actin caps could be a result of excessive membrane ruffling, which is induced by growth factor stimulation of PI3K and PIP3 (Rodriguez-Viciana et al., 1997; Deming et al., 2008; Yan et al., 2024).

To measure myosin recruitment directly, we live-imaged a GFP-fusion protein of the regulatory light chain of non-muscle myosin II (sqh:GFP), which localizes to regions of active myosin contractility, in control and *gcl−/−* embryos. During nuclear cycle 10, as in somatic syncytial buds, non-muscle myosin II initially formed a diffuse network occupying the cortex between pole buds (Foe et al., 2000; Zhang et al., 2018). About 150 s before nuclear cycle 10 metaphase, myosin II organized into ring structures at the base of the pole buds as they began to constrict in control *gcl*+*/−* embryos while remaining open in *gcl−/−* embryos. The membrane-to-cytoplasm ratio of myosin II fluorescence is higher in *gcl*+*/−* pole buds compared with *gcl−/−* pole buds, reflecting the direct connection between myosin assembly and pole bud constriction (Fig. 8, D–F). The failure of myosin II to localize to the cleavage furrow is consistent with observations in *pten−/− Dictyostelium discoideum* cells, which exhibit ectopic PIP3 production and defective cytokinesis (Janetopoulos et al., 2005).

If myosin II recruitment is inhibited when PIP3 levels are high, we would expect that even in WT embryos, myosin II recruitment is decreased at the membrane where Torso RTK activity is high. Indeed, consistent with the spatial patterning of Torso RTK, we found that in fixed embryos, myosin II fluorescence intensity is decreased at the anterior pole and the regions adjacent to the posterior pole, but is increased at the posterior pole, where germplasm is recruited, during PGC formation (Fig. 8, G–I).

**Figure S5.**
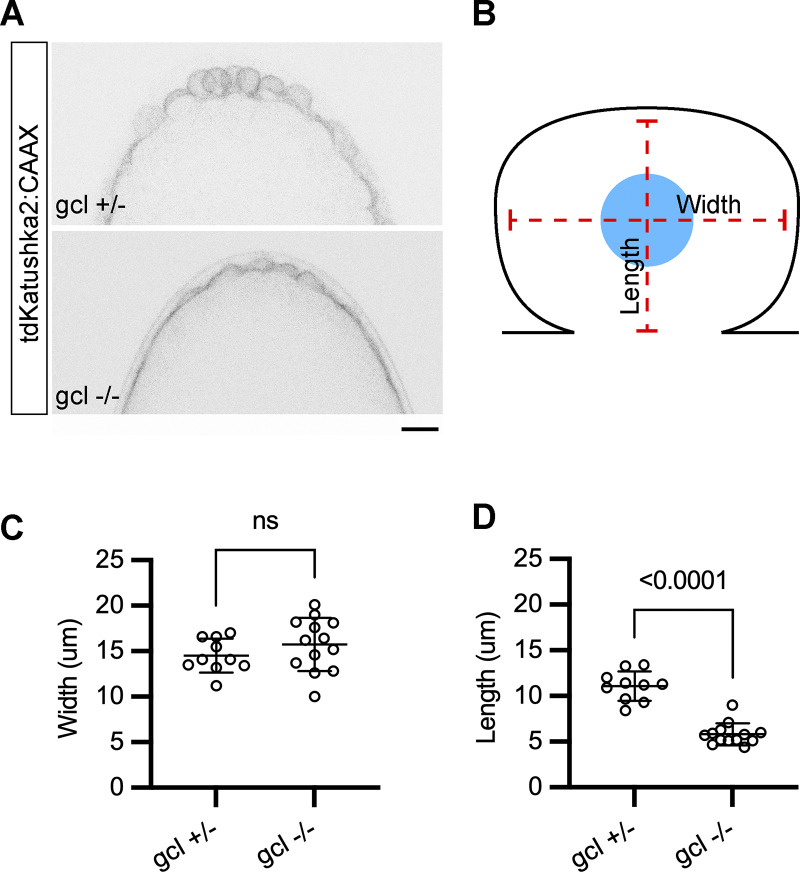
**Pole bud membrane shape in embryos from *gcl−/−* mothers is flattened due to an imbalance in phosphoinositides. (A)** Nuclear cycle 10 embryos from *gcl*+*/−* and *gcl−/−* mothers also expressing the membrane marker tdKatushka2:CAAX were live imaged on their side. These pole buds are tightly clustered together but have not cellularized. Scale bar = 20 µm. **(B)** Diagram of pole bud length and width measurements. **(C)** The width of pole buds was measured and plotted. Bars represent the mean ± standard deviation. (*n* = 10 buds from embryos derived from *gcl*+/− mothers, *n* = 13 buds from embryos derived from *gcl−/−* mothers, Mann–Whitney test). **(D)** The length of pole buds was measured and plotted. Bars represent the mean ± standard deviation. (*n* = 10 buds from embryos derived from *gcl*+*/−* mothers, *n* = 13 buds from embryos derived from *gcl−/−* mothers, Mann–Whitney test).

These observations suggest a model in which decreased actomyosin contractility may influence the rounding of pole buds. We measured the height and width of pole buds during cellularization using the membrane marker Katushka2-CAAX in both *gcl*+*/−* and *gcl−/−* embryos. Our findings indicate that the width of pole buds is similar in *gcl−/−* embryos compared with the control, but the height is reduced in *gcl−/−* embryos. Pole buds extend less from the embryo cortex and exhibit a flatter shape in *gcl* mutants (Fig. S5, A–D). This flat phenotype is consistent with cells lacking PTEN and exhibiting heightened PIP3 levels (Tang et al., 2011), indicating that successful pole bud formation depends on the spatiotemporal regulation of actomyosin contractility through the antagonism between GCL and Torso at the posterior pole.

## Discussion

Here, we describe the role of Torso and its suppressor GCL in establishing a spatial asymmetry in PI3K activation and PIP3 production, which is crucial for proper PGC formation. While Torso’s role in activating the transcription of terminal genes in *Drosophila* is well established, linking Torso RTK to PI3K activity is unexpected. Our study reveals an early function of Torso RTK activity in the spatial patterning of cortical PIP3 through PI3K activation, which occurs before activation of the Raf/MEK signaling pathway. During nuclear cycle 9–10, nuclei migrate to the cortex, and it is now clear that the membrane they reach already varies in its lipid composition. This also highlights an earlier role of GCL in “preparing” the cortex prior to the arrival of the syncytial nuclei. Once nuclei reach the posterior pole, GCL is recruited to their nuclear envelopes through its NLS, serving as a means to sequester it and prevent further activity (Pae et al., 2017). Since the nuclear localization of GCL is not necessary for its role in protecting PGC formation from Torso RTK (Pae et al., 2017), we propose that GCL’s role in patterning the posterior pole membrane is completed by the time the nuclei arrive.

By targeting Torso for degradation, GCL prepares a membrane domain with low PIP3 levels, thereby facilitating PGC formation in the subset of nuclei that reach the posterior pole. When GCL is not maternally inherited, *gcl−/−* embryos exhibit high PIP3 levels at the posterior pole membrane. Pole buds originating from this PIP3-rich membrane cannot recruit sufficient myosin II for timely bud furrow constriction, preventing PGC formation. Since PIP2 levels are unaffected in *gcl−/−* embryos, we speculate that this observed decrease in myosin II recruitment is most likely due to PIP3-dependent activation of factors that deplete actomyosin activity, such as the ArfGEF Steppke and atypical PKC (aPKC) (Lee and Harris, 2013; David et al., 2013; Ivey et al., 2014; Lee et al., 2015).

It has been suggested that germplasm presence facilitates outward budding produced by polymerization of a branched F-actin network through PIP2 enrichment, although the regulator of this enrichment has not been identified (Kilwein et al., 2025). Asymmetric actomyosin contractility is crucial for establishing polarity in various contexts, such as the early *Caenorhabditis elegans* embryo, where myosin recruitment is inhibited at the posterior pole due to an unknown centrosome-dependent polarity cue. The anterior bias of the actomyosin network correlates with an enrichment of PIP2 cortical structures and depends on the asymmetric distribution of PAR proteins (Scholze et al., 2018). However, further studies revealed that this could be attributed to an asymmetric membrane topology and differential labeling by the membrane probes used (Hirani et al., 2019). Further experiments are needed to show whether a similar asymmetric membrane topology could explain the recently reported posterior enrichment of the PIP2 probe (Kilwein et al., 2025). PIP3 levels are highly tuned to growth factor signaling, while PIP2 levels are not. Since our probes show no difference in PIP2 levels in *gcl*+/− and *gcl−/−* pole buds (Fig. 6, B–D), the observed increase in PIP3 synthesis in *gcl−/−* embryos likely results from heightened PI3K activity rather than a simple increase in plasma membrane bunching at the posterior pole. Our study now links the distribution of PIP3 directly to GCL’s action and a restriction of PI3K activity, since it is apparent that even *gcl*+/− pole buds carry more PIP3 than their WT counterparts, despite making the same number of PGCs (Fig. 7 C). Thus, PGC formation depends on the proper recruitment and spatial distribution of these lipid secondary messengers and their subsequent ability to regulate myosin assembly.

The posterior PIP3-rich membrane domain surrounding germplasm may play a role in limiting the number of PGCs that form, as our findings indicate that inhibiting Ras and PI3K activity causes the emergence of ectopic PGCs, akin to when GCL is overexpressed (Jongens et al., 1994). Ectopic PGCs in embryos overexpressing GCL lack sufficient germplasm factors necessary for survival and specification, resulting in their death before they reach the gonad (Jongens et al., 1994). It may be energetically undesirable for the embryo to generate more germ cells than it can sustain, and the band of PIP3-rich membrane encircling the posterior pole could be one mechanism ensuring that germ cell formation aligns with the available germplasm.

PIP3 levels must be tightly regulated in both space and time to allow cytokinesis to complete, primarily through the opposing behaviors and distinct subcellular localizations of PI3K and PTEN (Janetopoulos et al., 2005). Previous work on a PTEN mutant germline in *Drosophila* revealed defects in germplasm organization during oogenesis and nuclear migration during early embryogenesis, which obscured PTEN’s effects on PGC formation (von Stein et al., 2005). Our findings reveal an additional hierarchy of regulation in the early *Drosophila* embryo that employs the selective degradation of an RTK to limit PI3K activity and PIP3 production at the posterior pole. PIP2 and PIP3 levels are known to be important for somatic cellularization in *Drosophila*, and the ability to finely balance myosin assembly and disassembly is essential for furrow growth and constriction later in embryogenesis (Reversi et al., 2014). Outside of the posterior pole, the syncytial buds of the early embryo that give rise to the soma require a balance between actin polymerization and actomyosin contractility to ensure proper swelling (Fernandez-Gonzalez and Harris, 2023). Our results demonstrate that a similar equilibrium is necessary for the proper cellularization of posterior pole buds. Failure of myosin recruitment, signaled by PIP3 overproduction, results in flattened pole buds that cannot pinch off from the rest of the embryo. These findings enhance our understanding of this asymmetric cellularization event and the precise coordination of the cytoskeletal machinery required.

It remains unclear whether the creation of Torso-dependent PIP3 domains at the termini of the early embryo has a functional role or is simply a consequence of the initial partitioning of activated Torso during oogenesis, which is necessary to ensure the specification of the terminal regions. *Torso*^*HH/WK*^ embryos do not exhibit any notable defects at nuclear cycle 14, except for the “pole hole,” a cellularization defect at the posterior where germ cells form (Perrimon et al., 1985). Similarly, the overproduction of PIP3 at the posterior pole of *gcl−/−* embryos does not significantly affect nuclear migration or somatic cellularization, but does impair the ability of pole buds to cellularize. Therefore, it can be argued that the interaction between Torso and GCL is essential for establishing a soma-germline border by restricting lipid distribution in the embryonic cortex.

## Materials and methods

### *D. melanogaster* maintenance and genetics

*D. melanogaster* were raised at 25°C on medium containing 1.5% yeast, 3.6% molasses, 3.6% cornmeal, 0.112% Tegosept, 1.12% alcohol, and 0.38% propionic acid. Apple juice plates for embryo collection contained 25% apple juice, 2.5% sucrose, 2.25% bactoagar, and 0.15% Tegosept.

PGC formation is entirely controlled by maternal factors contributed to the embryo and independent of the paternal genotype. Additionally, PGC formation occurs independently of zygotic genome activation, and zygotic transcription has been shown to be dispensable for PGC formation. Therefore, unless otherwise specified, all genetic crosses are designed so that the mother expresses the respective transgene, construct, or mutant genotype. Throughout the text, “mutant embryo” refers to an embryo derived from a genotypically mutant mother or a mother carrying and expressing the respective transgene. For example: “*gcl−/−* embryo” refers to an embryo derived from a homozygous mutant *gclΔ/ gclΔ* mother, while “*gcl*+/− embryo” refers to an embryo derived from a *gclΔ/*+ mother carrying the mutant allele and either a balancer or WT chromosome. The maternal inheritance patterns are described more fully in the respective figure legends. All fly lines used are listed in the reagent table.

### Histology

For embryo collection, young parents (less than 5 days after eclosure) were transferred to embryo collection cages equipped with yeasted apple juice plates. To optimize embryo collection in the late morning, cages were kept in incubators set to a 10 pm–10 am light-dark cycle. To collect nuclear cycle 13–14 embryos for PGC counting, flies were allowed to lay eggs for two h at 25°C. Then the plates were removed, and the embryos were aged for an additional hour before fixation.

Embryos for PGC counting were dechorionated with 50% bleach for two min and fixed for 15–20 min in a biphasic solution of heptane and 4% PFA-1xPBS on a shaker. The aqueous layer was removed, and 10 ml of methanol was added for de-vitellenization followed by 1 min of robust shaking by hand. Fresh methanol was exchanged three times before storing at −20°C or continuing with the immunofluorescence staining protocol. Blocking and staining were carried out in PBS/0.1% BSA/0.1% Triton-X-100.

For myosin II quantification, embryos were dechorionated with 50% bleach for 2 min and fixed for 1 h in a biphasic solution of heptane and 4% PFA-1xPBS on a shaker, then manually devitellinized as follows: the PFA phase was removed, and embryos were rinsed twice briefly with PBS. Using a glass Pasteur pipette, embryos were transferred to plastic Petri dishes and briefly dried to facilitate adhesion to the plastic surface. The embryos were then covered in PBS, and under a dissecting stereomicroscope, manually removed from their vitelline membranes using a fine syringe. PBS/0.3% Triton-X-100 was added to the dish to free the devitellinized embryos from the plastic, and the embryos were transferred to a microfuge tube and washed five times in PBS/0.3% Triton-X-100 to remove all traces of PFA before continuing with the immunofluorescence staining protocol.

### Immunofluorescence

The wash buffer used was PBS/0.1% Triton-X-100 (PBST). All wash steps were performed while embryos rotated periodically on a nutator. Methanol-devitellinized embryos were gradually rehydrated with 3:1, 1:1, and 1:3 methanol:PBST, then washed three times with PBST before a 30-min block with 1% BSA in PBST. Embryos were incubated in primary antibody in blocking buffer overnight at 4°C, using the concentrations listed on the reagents table, and then washed five times with PBST at room temperature. Embryos were blocked again for 30 min. They were then incubated in secondary antibody in blocking buffer for 4 h at room temperature in the dark, followed by three washes with PBST. Lastly, embryos were equilibrated in VECTASHIELD (Vector Laboratories) overnight at 4°C before mounting on coverslips and imaging.

Immediately after fixation, hand-devitellinized embryos were blocked for 30 min in PBS/1% BSA/0.3% Triton X-100, and then counterstained overnight with 1:1,000 Phalloidin and 1:2,000 DAPI (2 mg/ml). Phalloidin (F-actin) and DAPI (DNA) were used for staging embryos. The embryos were washed five times in PBS/0.3% Triton-X-100, then allowed to equilibrate overnight in VECTASHIELD mounting media. The following day, the embryos were mounted on glass slides and imaged.

Imaging of fixed embryos was done on an inverted Zeiss LSM 780 laser scanning confocal microscope with a Zeiss air Plan-Apochromat 20×/0.8 objective.

### Plasmids

To generate UASp-Ras, the Ras coding sequence was amplified from a cDNA clone ordered from DGRC and cloned into a pWallium22 vector. To generate UASp-RalA-nosTCEpgc 3′UTR, UASp-Rgl-nosTCEpgc 3′UTR, UASp-PI3K92E-nosTCEpgc 3′UTR, and UASp-Pi3K21B-nosTCEpgc 3′UTR lines, the coding sequence of the gene of interest was amplified from cDNA clones ordered from DGRC and cloned into a pWallium22 vector containing the PGC-specific 3′UTR (TCEp3), provided by Dr. Benjamin Lin. All constructs were integrated into attp2, attp40, and attp5 by BestGene. A full list of plasmids is included in the reagents table. Missense mutants were generated by Q5 site-directed mutagenesis kit (New England Biolabs) according to the manufacturer’s protocol using primers designed through NEBaseChanger.

### Time-lapse imaging of PGC formation

To capture PGC formation, embryos were collected for 1.5 h. Embryos were dechorionated with 50% bleach for 2 min. Heptane glue was made by dissolving tape adhesive into heptane. To mount for imaging, embryos were glued either on their sides or their posterior poles to 35-mm coverslip dishes (MATTEK P35G-1.5-14-C) and submerged with a layer of halocarbon oil 700 (#H8898; Sigma-Aldrich). Images were acquired on an inverted Nikon Ti2 with a Yokogawa CSU W1 spinning disk scanhead and Kinetix camera, using a Nikon Plan Apo Lambda D DIC N2 60×/1.42 oil objective controlled by Nikon Elements. For posterior-mounted embryo time-lapse movies, stacks of 20-µm thickness with 1-µm step size were acquired every 30 s. All stacks begin at the coverslip to capture the entire membrane of the pole buds.

Images for pole bud height and width measurements (Fig. S3) were acquired on an upright LSM 980 laser scanning confocal microscope with a Zeiss air Plan-Apochromat 20×/0.8 objective. To mount for imaging on an upright scope, embryos were glued on their sides to a coverslip and placed on a gas-permeable membrane (#94.6170.102; SARSTEDT) that was covered with halocarbon oil 27 (#H8733; Sigma-Aldrich).

### Optogenetic activation of Sos

Transgenic flies carrying the OptoSos construct were kindly provided by the Toettcher laboratory (Johnson et al., 2017). Embryos were collected on apple juice plates for 30 min, dechorionated in 50% bleach for 2 min, thoroughly rinsed in water, and then mounted on coverslips using heptane glue. Embryos were imaged and activated using an inverted Zeiss LSM 780 laser scanning confocal microscope with a Zeiss air Plan-Apochromat 20×/0.8 objective. Light (488 nm) was applied at 40% power for 10 iterations every 30 s starting 10 min after collection for 100 min.

### PIP3/PIP2/F-actin/myosin II pole bud measurements

Fluorescence intensity analysis of PIP3, PIP2, F-actin, and myosin II in time-lapse images of posteriorly mounted live embryos (Fig. 6, C and F; Fig. 7, C and D; and Fig. 8, B and E) was performed using custom Python scripts. Prior to segmentation, the nuclear marker channel of the two-channel z-stack fluorescence images was denoised using a Noise2Void (Krull et al., 2019) model to enhance signal-to-noise ratio, trained from images from time points not included in the final analysis. Subsequently, pole buds were segmented in 3D using a Cellpose-SAM model (Pachitariu et al., 2025, *Preprint*), and nuclei were segmented using a Cellpose cyto3 model (Stringer and Pachitariu, 2025). Both models were trained following a human-in-the-loop approach (Pachitariu and Stringer, 2022) using random xy slices from the datasets and 3D segmentations generated using the stitching mode in Cellpose.

The resulting 3D masks for pole buds and nuclei were then processed to define three distinct cellular compartments: nucleus, cytoplasm, and cell membrane. The nuclear compartment was directly defined by the nucleus mask. To define the pole bud periphery for membrane analysis, whole pole bud masks underwent subtraction of a 3D erosion operation from a dilation operation, both using a ball structuring element. This peripheral mask was further refined by thresholding, retaining only pixels with an intensity in the PIP3/PIP2/F-actin/myosin II channel >105% of the mean cytoplasmic intensity. The mean cytoplasmic intensity for this refinement step was measured in a region defined by dilating the nucleus mask by two pixels (0.2 µm). The cytoplasm mask was generated by eroding the whole cell mask and subsequently subtracting the nuclear mask.

For each segmented pole bud, mean PIP3/PIP2/F-actin/myosin II intensities within the nucleus, cytoplasm, and cell membrane masks were calculated from raw images without additional preprocessing. Intensity ratios between membrane and cytoplasm were determined.

Figures and plots were generated using ImageJ and GraphPad Prism. The Mann–Whitney U test was used on GraphPad to compare data sets. Every time point from the time-lapse was processed using the method described above.

### PGC counting

Fixed embryos were stained with anti-Vasa antibody and DAPI to visualize pole cells and DNA. Only embryos that were at nuclear cycle 13–14, prior to somatic cellularization, were counted. Multiple z-planes with a 2.5-µm step size encompassing the area of PGC formation were imaged on an inverted Zeiss LSM 780 laser scanning confocal microscope with a Zeiss air Plan-Apochromat 20×/0.8 objective. Vasa-positive PGCs in each embryo were counted manually using the ImageJ Cell Counter plugin (NIH; http://rsb.info.nih.gov/ij/) (Schindelin et al., 2012). The Mann–Whitney U test was used on GraphPad to compare data sets.

### Myosin II quantification in fixed embryos

The following imaging and analysis protocol was performed on hand-devitellinized embryos expressing sqh-sqh:mScarlet. Single z-slices containing the middle Z-plane of each embryo were acquired on a Zeiss 780 point scanning confocal microscope with 2× averaging, 16-bit depth and 2048 × 2048 image size using the 561-nm laser line with 0.6× zoom. Zeiss Air Plan-Apochromat 20×/0.8 objective was used. Images were then manually rotated to align the A-P axis horizontally.

To quantify myosin signal profiles across the embryo circumference, masks of the myosin signal were segmented and intensity was quantified using a custom ImageJ macro as follows. First, cleavage furrows were emphasized using the find edges function; then, a pixel-maximum filter with a 3-pixel radius was applied. Next, a binary threshold was manually applied to create a mask that encompassed the embryonic cleavage furrows. Gaps in the detected contours of the embryo circumference were manually filled to produce a single, continuous mask representing the embryo circumference. The ROI corresponding to the mask was then transferred to the original unaltered image for quantification. The ROI was radially divided into 100 equal-angle segments, and the mean myosin signal intensity for each segment was quantified. These data were then processed in R to normalize each segment’s average myosin signal intensity to the average myosin signal across the entire ROI for each embryo, which allowed for combining myosin intensity profiles from multiple embryos in a single plot. Plots were generated using the base plotting system in R.

### Online supplemental material

Fig. S1 is related to Fig. 2 and shows that maternal knockdown of *csw*, *dsor*, and *rolled* fails to rescue PGC defect in embryos from *gcl−/− mothers*. Fig. S2 is related to Fig. 3 and demonstrates that increasing RalA activity only mildly antagonizes PGC formation. Fig. S3 is related to Fig. 5 and describes the uniform distribution of p60, the regulatory subunit of PI3K, in *Drosophila* across the embryo prior to PGC formation. By nuclear cycle 14, p60 localizes to furrows and is excluded from pole cells. Fig. S4 is related to Fig. 7 and illustrates that PIP2 and PIP3 occupy separate membrane compartments of the embryo and the posterior pole. Fig. S5 shows that the pole bud membrane shape in embryos from *gcl* mutant mothers is flattened due to an imbalance in phosphoinositides. Video 1 relates to Fig. 7 and Fig. S4 and depicts PIP2 and PIP3 membrane compartments in a laterally mounted embryo. Table S1 lists fly stocks and reagents used. Video 1 (related to Fig. 7 and Fig. S4) shows PIP2 and PIP3 membrane compartments in a laterally mounted embryo.

## Supplementary Material

Review History

Table S1 shows a reagent and resource table.

## Data Availability

The data underlying all figures are available in the published article and supplemental information, and from the corresponding author.
