# Supplementary material for: GCL pruning of PIP3 establishes the soma-germline boundary
Source: J Cell Biol. 2026 Jul 22;225(9):e202604036. doi: 10.1083/jcb.202604036 (PMC13390627; doi:10.1083/jcb.202604036)
Supplement: Table S1 — shows a reagent and resource table. [file jcb_202604036_tables1.docx]

**Reagent and Resource Table**

| **REAGENT or RESOURCE** | **SOURCE** | **IDENTIFIER** |
| --- | --- | --- |
| Fly Stocks | | |
| *w^1118^* | Bloomington | BL #3605 |
| *gclΔ*/CyO (referred to as *gcl+/-* in text) | T. Jongens | N/A |
| maternal-alphaTub-GAL4-VP16 | Bloomington | BL #7063 |
| maternal-alphaTub-GAL4-VP16 | Bloomington | BL #7062 |
| alphaTub-GAL4-VP16 | Bloomington | BL #5138 |
| w^-^;; nos-Lifeact:TdTomato-p2A tdKatushka2:CAAX-nos3’utr (VK27) | B. Lin | Lin et al., 2022 |
| w^-^;; His2Av:mRFP1 | Bloomington | BL #23650 |
| w^-^;; His2Av:GFP | Bloomington | BL #5941 |
| UASp-OptoSos (tRFP-SSPB-SOScat-P2A-iLID-CAAX) | J. Toettcher | (Johnson et al., 2017) |
| w^-^;; UASp-mClover2-nosTCEpgc 3’utr (attp2) | B. Lin | N/A |
| w^-^; UASp-mClover2:Rgl-nosTCEpgc 3’utr (attp2) | This study | N/A |
| w^-^; UASp-mClover2:RalA-WT-nosTCEpgc 3’utr (attp2) | This study | N/A |
| w^-^; UASp-mClover2:RalA-S25N nosTCEpgc 3’utr (attp2) | This study | N/A |
| w^-^; UASp-mClover2:RalA-G20V nosTCEpgc 3’utr (attp2) | This study | N/A |
| w^-^; UASp-EGFP:Ras-WT (RNAi Resistant) attp40; ras RNAi attp2 | This study | N/A |
| w^-^; UASp-EGFP:Ras-C40 (RNAi Resistant) attp40; ras RNAi attp2 | This study | N/A |
| w^-^; UASp-EGFP:Ras-G37 (RNAi Resistant) attp40; ras RNAi attp2 | This study | N/A |
| w^-^; UASp-EGFP:Ras-S35 (RNAi Resistant) attp40; ras RNAi attp2 | This study | N/A |
| w^-^;; His2Av:mRFP1, sqh-GFP:moe[ABD] | Bloomington | BL #59023 |
| w^-^;; sqh-sqh:3xGFP | Y. Bellaïche | Pinheiro et al., 2017 |
| sqh-sqh-mscarlet (X) | Bloomington | BL #94929 |
| w^-^;; Ubi-PLCγ[PH]:mCherry | Y. Bellaïche (gift from T. Harris) | Herszterg et al., 2013 |
| w^-^; alphaTub84B-GFP:Grp1[PH] | Bloomington | BL #8163 |
| w^-^;; alphaTub84B-GFP:Grp1[PH] | Bloomington | BL #8164 |
| UASp-mcherry RNAi (attp2) | Bloomington | BL #35785 |
| UASp-pi3k92e RNAi GL00311 (attp2) | Bloomington | BL #35798 |
| UASp-torso RNAi GLV21002 (attp2) | Bloomington | BL #35639 |
| UASp-torso RNAi HMS00021 (attp2) | Bloomington | BL #33627 |
| UASp-torso RNAi GL00222 (attp2) | Bloomington | BL #35316 |
| w^-^;; UASp-shc RNAi (attp2) | This study | shRNA: CAGCAACCTCATCGACCTCAA |
| UASp-sos RNAi HMS00149 (attp2) | Bloomington | BL # 34833 |
| w^-^;; UASp-ras RNAi (attp2) | This study | shRNA: GCCGAATCGTAGATTTAAATG |
| w^-^;; UASp-ras RNAi (attp2) | This study | shRNA: GCAAGTGGTTATCGATGGAGA |
| UASp-csw RNAi GL00095 (attp2) | Bloomington | BL #35215 |
| UASp-csw RNAi GLV21001 (attp2) | Bloomington | BL #35638 |
| UASp-raf RNAi (attp2) | This study | shRNA: CACGAGCACCTTGAAACACAA |
| UASp-ksr RNAi GL01134 (attp2) | Bloomington | BL #41598 |
| UASp-dsor1 RNAi HMS00145 (attp2) | Bloomington | BL #34830 |
| UASp-dsor1 RNAi GLV21003 (attp2) | Bloomington | BL #36099 |
| UASp-rolled RNAi HMS00173 (attp2) | Bloomington | BL #34855 |
| UASp-rolled RNAi GL00215 (attp2) | Bloomington | BL #36058 |
| UASp-PI3K-reg:HA (VK37) | J. Zallen | Tamada et al., 2022 |
| UASp-PI3K-reg-ΔSH2:HA (VK37) | J. Zallen | Tamada et al., 2022 |
| w^-^;; UASp-PI3K21B:HA-nosTCEpgc 3’utr (attp2) | This study | N/A |
| w^-^;; UASp-HA:PI3K92E-nosTCEpgc 3’utr (attp2) | This study | N/A |
| w^-^;; UASp-HA:PI3K92E-CAAX-nosTCEpgc 3’utr (attp2) | This study | N/A |
| w^-^;; UASp-HA:PI3K92E-ΔSH2 nosTCEpgc 3’utr (attp2) | This study | N/A |
| w^-^;; UASp-HA:PI3K92E-D954A nosTCEpgc 3’utr (attp2) | This study | N/A |
| p60-fosmid-2XTY1-SGFP-V5-preTEV-BLRP-3XFLAG | Vienna Drosophila Resource Center | v318864 |
| *torso^HH^*/cyo | T. Schüpbach | N/A |
| *torso^WK^*/cyo | T. Schüpbach | N/A |
| *st^1^ tsl^3^*/TM3, Ser | Bloomington | BL #3310 |
| *e^1^ tsl^4^*/TM3, Ser | Bloomington | BL #3289 |
| Antibodies | | |
| Rabbit polyclonal anti-Vasa (1:5000) | R. Lehmann | N/A |
| Mouse monoclonal anti-HA (1:500) | Enzo | ENZ-ABS120 |
| Chicken polyclonal anti-GFP (reacts with mClover2) (1:500) | Rockland | 600-901-215 |
| Anti-chicken AF488 (1:500) | Jackson Biolabs | 703-545-155 |
| Anti-rabbit Cy3 (1:500) | Jackson Biolabs | 711-165-152 |
| Anti-mouse CF488 (1:500) | Biotium | 20014-1mg |
| Rabbit monoclonal anti-dpERK (1:250) | Cell Signaling | 4370 |
| Alexa Fluor™ 488 Phalloidin (1:1000) | ThermoFisher | A12379 |
| DAPI (2mg/mL) | ThermoFisher | D1306 |
|  |  |  |
| Recombinant DNA | | |
| pWallium22 | Drosophila Transgenic RNAi Project |  |
| cDNA: Ras LD17536 | Drosophila Transgenic RNAi Project | DGRC #4508 |
| cDNA: RalA LD21679 | Drosophila Genomics Resource Center | DGRC #5793 |
| cDNA: Rgl LD16082 | Drosophila Genomics Resource Center | DGRC #5074 |
| cDNA: Pi3K92E SD05105 | Drosophila Genomics Resource Center | DGRC #5287 |
| cDNA: Pi3K21B LD42724 | Drosophila Genomics Resource Center | DGRC #5517 |
| Chemicals | | |
| Vectashield Antifade Mounting Medium | Vector  Laboratories | Cat# H-1000,  H-1200 (with  DAPI) |
