# Supplementary material for: GCL pruning of PIP3 establishes the soma-germline boundary
Source: J Cell Biol. 2026 Jul 22;225(9):e202604036. doi: 10.1083/jcb.202604036 (PMC13390627; doi:10.1083/jcb.202604036)
Supplement: Review History [file jcb_202604036_review_history.pdf]

GCL pruning of PIP3 establishes the soma-germline boundary

Mariyah Saiduddin, Juhee Pae, Asier Marcos-Vidal, Martin Alani, and Ruth Lehmann

Corresponding Author(s): Ruth Lehmann, Whitehead Institute for Biomedical Research

**Review Timeline:**	Submission Date:	2026-04-06
	Editorial Decision:	2026-05-04
	Revision Received:	2026-06-04

Monitoring Editor: Julius Brennecke

Scientific Editor: Gabriele Stephan

Transaction Report:

(Note: With the exception of the correction of typographical or spelling errors that could be a source of ambiguity, letters and reports are not edited. The original formatting of letters and referee reports may not be reflected in this compilation.)

DOI: https://doi.org/10.1083/jcb.202604036

Revision 0

Review #1

1. Evidence, reproducibility and clarity:

Evidence, reproducibility and clarity (Required)

This is an outstanding and elegant study that addresses an important question in developmental and germline biology: how the soma-germline boundary is established during embryogenesis. This represents one of the most fundamental cell-fate decisions during organismal development. The authors combine elegant genetics, optogenetics, quantitative live imaging, and lipid biosensors to provide a compelling mechanistic framework linking receptor degradation, lipid signaling, and cytoskeletal dynamics. They show that Torso signaling via PI3K and PIP3 antagonizes primordial germ cell (PGC) formation and promotes somatic cell fate. Furthermore, they demonstrate that GCL-mediated degradation of Torso at the posterior pole creates a PIP3-low membrane domain that permits myosin II recruitment and pole bud constriction, thereby enabling PGC formation. Together, these results clearly demonstrate how the soma-germline boundary is established.

We have only minor comments on the manuscript, primarily aimed at improving clarity for non-specialist readers:

1. Figure 1D: It would be useful to indicate the number of embryos analyzed for these experiments (n = ?).
2. Figure 3B: The control condition for $gcl^{-}/^{-}$; ras-RNAi is labeled as "EV". This terminology (presumably "empty vector") is not defined in either the text or the figure legend. In addition, the magenta channel for the Ras-G37 condition appears to be flipped horizontally.
3. Page 7: The text states that "Ras-C40 activates the PI3K pathway," whereas the figure depicts Ras-C40 as activating the RalA pathway. This discrepancy could be confusing for the reader and should be corrected.
4. Figures 4 and 5: To facilitate interpretation, it may be helpful to include a schematic of the PI3K complex indicating the different subunits used in the study, along with information (potentially color-coded) about whether each construct primarily acts as an activator or inhibitor of PI3K function.
5. Figure 4A and 4B: For clarity and consistency with the text, the panels (and corresponding plots) for dp110-WT and dp110-CAAX could be placed before those for dp110-D954A and dp110-ΔRBD.

6. Figure 5C: The term "p60-TCEp3," which appears to correspond to the germ plasm-targeted p60-WT construct, is not defined in either the figure legend or the main text.
7. Page 12: The reference "(Fig. S1A, Movie 1)" should be corrected to "(Fig. S2A, Movie 1)."
8. Page 13: There is a missing word in the sentence "the biosensor appeared to be enrich to...", which should be corrected to "enriched."
9. Figure 7A: Although the data presented are interesting and ultimately support the authors' conclusion that Torso regulates PIP3 levels, the results are somewhat counter-intuitive and may be confusing for readers. The authors might consider moving this panel to the Supplementary Figures. In addition, it could be informative to include PIP3 measurements for $gcl^{-}/^{-}$ (and possibly $gcl^{+}/^{-}$) pole buds in Figure 7B, as PIP3 appears particularly enriched in these conditions compared to wild type.

2. Significance:

Significance (Required)

The biological question is highly interesting, the experimental design is very clear, and the data are convincing throughout. The imaging, quantification, and movies are of very high quality and strongly support the authors' conclusions. Overall, this manuscript represents a significant conceptual and technical advance and will be of broad interest to the fields of germline biology, membrane biology, and embryonic morphogenesis.

3. How much time do you estimate the authors will need to complete the suggested revisions:

Estimated time to Complete Revisions (Required)

(Decision Recommendation)

Less than 1 month

4. *Review Commons* values the work of reviewers and encourages them to get credit for their work. Select 'Yes' below to register your reviewing activity at [Web of Science Reviewer Recognition Service](#) (formerly Publons); note that the content of your review will not be visible on Web of Science.

Web of Science Reviewer Recognition

Yes

Review #2

1. Evidence, reproducibility and clarity:

Evidence, reproducibility and clarity (Required)

The MS by Saiduddin et al. investigates the connection between the germ cell-less (gcl) germplasm component, the RTK Torso and cortical PI3P levels in the formation of a normal complement of primordial germ cells (PGCs) in the Drosophila embryo. The authors find that GCL regulates Torso levels, which in turn activate the PI3-kinase in a ras-dependent (but raf-independent) manner. It then follows that the realm of action of gcl defines a region at the posterior pole of the embryo where PI3P levels are sufficiently low to allow PGC formation.

Specific points:

- Fig. legends to 1C and 1D are swapped.
- Why is csw not necessary for PGC formation? It acts upstream of Ras. This is not discussed.
- Fig 3C. Consider changing the order of the ras-variants used: S35, G37, C40 instead of S35, C40, G37.
- Fig 4A, B: Consider changing the order of the panels. Control, dp110-wt, dp110-CAAX, dp110-D954A, dp110-deltaRBD.
- Fig S4 is mentioned in the text before S2 and S3. Consider changing the suppl. figure order.
- Page 12: Fig S1 A does not show PIP2 dynamics. Movie 1 is not available to this reviewer. The authors most likely refer to fig. S2.
- Page 13, 1st para: Why do the authors use glc heterozygous embryos to look at PIP3 and PIP2? Particularly so when they report later in the MS that glc+/- behave differently to wt controls in terms of PIP3 levels (Fig. 7C). By looking at gcl+/+, they might find that now PIP2 levels are different in gcl mutant embryos or that the differences between PIP3 levels in +/+ and -/- are larger than compared with +/-.
- Pages 15 and 16: revise figure calls in the text.
- M+M: How were gcl+/- and gcl-/- embryos identified?

2. Significance:

Significance (Required)

This work reveals a novel, transcription-independent role for Torso in the regulation of cortical lipid compartmentalization and provides a molecular explanation for how Tor activity at the embryo posterior delimits the area where PGCs arise. The experiments are superbly documented, the MS is a pleasure to read and the hypotheses are elegantly tested. While the broader generality of the findings remains uncertain, particularly since

the role(s) of gcl in germ cell development do not seem to be evolutionary conserved, the study sheds light on a ras-dependent, transcription-independent function of RTKs in cellularization, a function most likely to be essential also in other contexts.

3. How much time do you estimate the authors will need to complete the suggested revisions:

Estimated time to Complete Revisions (Required)

(Decision Recommendation)

Between 1 and 3 months

4. *Review Commons* values the work of reviewers and encourages them to get credit for their work. Select 'Yes' below to register your reviewing activity at [Web of Science Reviewer Recognition Service] (formerly Publons); note that the content of your review will not be visible on Web of Science.

Web of Science Reviewer Recognition

No

Review #3

1. Evidence, reproducibility and clarity:

Evidence, reproducibility and clarity (Required)

Summary:

Although Torso is known to antagonize primordial germ cell (PGC) formation, the underlying mechanisms remain unclear. Canonical Torso signalling typically results in activation of Ras. However, the authors show that Ras-mediated suppression of PGC formation is independent of the Raf/MEK/ERK pathway. Instead, they uncover an unexpected role for Torso in activating phosphoinositide 3-kinase (PI3K) that promotes formation of PIP3 enriched posterior membrane domains. The resulting increase in PI3K activity disrupts PGC formation. Furthermore, they show that by promoting Torso degradation, the ubiquitin ligase adaptor Germ Cell-Less (GCL) primes the posterior membrane with reduced PIP3 to facilitate PGC formation. Lastly, the authors suggest a model where antagonistic relationship between GCL and Torso influences actomyosin contractility that may allow the bud to constrict for proper PGC formation.

Major comments:

Figure 1B: The authors describe that embryos with OptoSos still form buds which protruded from the cortex, but PGCs largely fail to cellularize (described in pg. 5). I'm not sure what they meant by "fail to cellularize" as this is not obvious to me when looking at the figure. The authors should describe how they know it's cellularized in the controls and not in the OptoSos or change the wording to "suggesting a failure to cellularize".

Figure 5C: The hyper-clustered phenotype they describe is hard to visualize in this figure (described in pg. 11). The authors should describe what is meant by "hyper-clustered".

Figure 6: In Fig. 6E-G, the "brightness" of PIP3 at the membrane corresponds to the images even with different views (posterior and orthogonal) and agrees with the graph.
However, when looking at Fig. 6B, it looks to me that PIP2 is brighter in gcl+/-, but the opposite is true when looking at Fig. 6D (i.e., PIP2 looks brighter in gcl-/-). The authors might want to comment on this.

It would also be helpful to show the overlap of the PIP2 and PIP3 signals in control vs. gcl mutants at different stages so the relative distribution and intensity of the signals can be better appreciated (consider adding this as a supplementary figure).

Figure 7: When comparing Fig. 7A and 7B torsoHH/WK images, we can see that in Fig. 7A that PIP3 pattern changes such that PIP3 is now at the most posterior end where PGC will eventually form (compared to control that has low PIP3 in this region), but then in Fig. 7B they are looking at the buds and they say PIP3 levels decrease, which does not correspond to Fig. 7A. Are these simply different stages and PIP3 levels change over time (looking at Fig. 7C, PIP3 does not seem to change a lot over time)?

Page 15, last paragraph: "If myosin II recruitment is inhibited when PIP3 levels are high"
Is it possible that inhibition of myosin II recruitment is due to conversion of PIP2 -> PIP3, thus loss of PIP2, or is it that myosin is specifically recruited to regions where PIP2 is high? This seems like a point that should be added to the discussion.

Overall, I think their claim that antagonistic activities of GCL and Torso is crucial for PGC formation is well justified. The combination of optogenetic tools with activation and lof mutants is nicely done. Some clarification regarding the PIP3 and PIP2 levels will be helpful to the reader (see my comments above). The myosin claim is less convincing (see my comment on Fig. 8D-F below).

Minor comments on the text:

p. 5, line 5: "Optosos" is written "OptoSos" elsewhere (suggest using OptoSos throughout)
p. 5, line 6: suggest adding a comma after "Ras" for clarity
p. 5, last line: the genotype is "w^1118" (with ^ indicating a superscript), not "w^-1118", and is italicized (this should be corrected throughout)
p. 6, line 2: replace "cellularizing" with "cellularization"
p. 6, lines 11-13: Where is it shown that knockdown of csw, dsor1 and rolled did not restore PGC formation? The data are not present in Fig. 2C (could include in supp fig?)
p. 7, line 1: replace "interfere" with "interferes"
p. 7, last three lines: what is stated here, "Ras-G37 [activates] both the RalA and the PI3K pathways, and Ras-C40 activates the PI3K pathway" is not consistent with what is diagrammed in Fig. 3C, where Ras-C40 is indicated as activating RalA (please correct either the text or the diagram)
p. 11, lines 1-2: the Pi3K21B gene and transcript should be italicized (note that Pi3K21B is the official gene name on FlyBase)
p. 11, lines 6-10: it might be helpful to explain how the p60 construct was overexpressed (current lines 9-10) before describing the results (current lines 7-8)
p. 12, paragraph 2, line 2: the PIP2 biosensor should be written as "PLCgamma[PH]:mCherry" throughout, not "PLCy[PH]:mCherry"; this should be changed in the figures as well as the text (Symbol font can be used to turn "g" into lower-case "gamma", both in Word and in Illustrator)
p. 12, paragraph 2, line 3: it does not appear that the two PIP markers were used "simultaneously" in Fig. 6A; however, this is evident from Fig. S2 and Movie 1 (consider placing callouts to these earlier in the paragraph or moving the description of simultaneous expression and observation of the two markers later in the paragraph to avoid confusion)
p. 12, paragraph 2, line 7: replace "Fig. S1A" with "Fig. S2" (this was confusing)
p. 16: change "Fig. 7G-I" to "Fig. 8G-I"
p. 20, Deming reference: there appears to be a stray asterisk in the title

Minor comments on the figures and figure legends:

Fig. 1B, lines 4-5: at what stage are these embryos? Cycle 9? Cycle 14? Both?
Fig. 1C: see previous comment about "w^1118" genotype nomenclature
Fig. 1D: need to explain that the colors in the graph indicate the numbers of PGCs formed (this could also be added as a label across the top of the graph); in addition, the number of embryos examined for each genotype should be included in the legend

Fig. 2B: spell out where csw, dsor1 and rolled data are shown; also, "n" is not defined; was this the number of embryos per genotype?
Fig. 3B: "EV" should be defined in the legend; is this "empty vector"?
Fig. 3C: see previous comment re: mistake in the diagram; I believe Ras-C40 was described as activating PI3K, not RalA
Fig. 3E: fix "w^1118" as described above
Fig. 4A: add dp110-CAAX results to Results section
Fig. 4B, line 2: was the graph plotted from the data in panel (C) or panel (A)? panel (A) seems more likely, because the data in C is plotted in D; please correct the panel callout
Fig. 5C: describe "p60-TCEp3" in the legend
Fig. 6A: define "(fire)" here or in the first figure legend where this is used
Figure 8 title: "Actin fluorescence is increased in gcl-/- pole buds",But their graph in Fig. 8B comparing actin in gcl+/- to -/- is not significant
Fig. 8D-F: Although the plots in panel E agree with the images in panel D, it is unclear why those in panel F are not more concordant. In F, myosin appears enriched at the cortex relative to the cytoplasm in gcl-/- mutants, which is hard to reconcile with the data in D-E.
Fig. 8I: replace "Scarlett" with "Scarlet"
Fig. S2A: define the three time points shown here, and clarify that these are shown left to right (if this is indeed the case)
Fig. S4: change "P60" to "p60" in the figure title

Movie: The movies showing PIP2 and PIP3 in whole embryos are nice, but it would also be helpful to also include merged images of the two channels, so the reader can examine the relative accumulation of the two PIPs over time.

Referees cross-commenting

I agree enthusiastically with the comments of the other reviewers, who often came to the same conclusion I did about the manuscript and the data, including some of the detailed points about the figures, etc.

2. Significance:

Significance (Required)

General assessment:

The many strengths of this manuscript include elegant genetic and optogenetic approaches using well-designed transgenes.

The main weakness is the lack of experiments showing simultaneous live imaging of the PIP2 and PIP3 sensors in gcl-/- and other genetic backgrounds, which would help the reader better envision how regulators of this pathway affect phospholipid distribution at the level of whole embryos and prospective pole cells. Note that because of the time required, I do not insist that they do this.

Advance:

Study demonstrates for the first time an unexpected role of Torso in PI3K regulation

Audience:

germ cell afficionados, developmental biologists, cell biologists, PI3K researchers

My field of expertise:

Drosophila, germ cell development, genetics, cell biology, live imaging, phosphoinositides

3. How much time do you estimate the authors will need to complete the suggested revisions:

Estimated time to Complete Revisions (Required)

(Decision Recommendation)

Between 1 and 3 months

4. *Review Commons* values the work of reviewers and encourages them to get credit for their work. Select 'Yes' below to register your reviewing activity at [Web of Science Reviewer Recognition Service] (formerly Publons); note that the content of your review will not be visible on Web of Science.

Web of Science Reviewer Recognition

Yes

Full Revision

Manuscript number: RC-xx-xx
Corresponding author(s): Ruth Lehmann

[Please use this template only if the submitted manuscript should be considered by the affiliate journal as a full revision in response to the points raised by the reviewers.

If you wish to submit a preliminary revision with a revision plan, please use our "Revision Plan" template. ***It is important to use the appropriate template to clearly inform the editors of your intentions.****]*

1. General Statements

We thank the reviewers for their thoughtful and in-depth comments. We have responded to all of the comments and made changes as suggested.

2. Point-by-point description of the revisions

Requested Revision	Response
**Reviewer #1**	
Figure 1D: It would be useful to indicate the number of embryos analyzed for these experiments (n = ?).	Number of embryos now included in figure legend
Figure 3B: The control condition for gcl⁻/⁻; ras-RNAi is labeled as "EV". This terminology (presumably "empty vector") is not defined in either the text or the figure legend. In addition, the magenta channel for the Ras-G37 condition appears to be flipped horizontally.	We replaced with "-" in figure and figure legend
Page 7: The text states that "Ras-C40 activates the PI3K pathway," whereas the figure depicts Ras-C40 as activating the RalA pathway. This discrepancy could be confusing for the reader and should be corrected.	The diagram has been corrected
Figures 4 and 5: To facilitate interpretation, it may be helpful to include a schematic of the PI3K complex indicating the different subunits used in the study, along with information (potentially color-coded) about whether each construct primarily acts as an activator or inhibitor of PI3K function.	Figure 4E and Figure 5E were added
Figure 4A and 4B: For clarity and consistency with the text, the panels (and corresponding plots) for dp110-WT and dp110-CAAX could be placed before those for dp110-D954A and dp110-ΔRBD.	Order of constructs was rearranged

Review COMMONS

Figure 5C: The term "p60-TCEp3," which appears to correspond to the germ plasm-targeted p60-WT construct, is not defined in either the figure legend or the main text.	Clarification was added to the text (p.11, line 225)
Page 12: The reference "(Fig. S1A, Movie 1)" should be corrected to "(Fig. S2A, Movie 1)."	Corrected
Page 13: There is a missing word in the sentence "the biosensor appeared to be enrich to...", which should be corrected to "enriched."	Corrected
Figure 7A: Although the data presented are interesting and ultimately support the authors' conclusion that Torso regulates PIP3 levels, the results are somewhat counter-intuitive and may be confusing for readers. The authors might consider moving this panel to the Supplementary Figures. In addition, it could be informative to include PIP3 measurements for $gcl^{-}/^{-}$ (and possibly $gcl^{+}/^{-}$) pole buds in Figure 7B, as PIP3 appears particularly enriched in these conditions compared to wild type.	We agree that at first the findings in the early embryos were confusing, but we prefer including them in the main figure to demonstrate changes in PIP 3 distributions in torso mutants. We are now providing a possible explanation for these findings (p13 line 270-). The differences are quite clear in the older embryos and measurements shown in 7B-D. Pole bud measurements for *gcl-/-* and *gcl+/-* are shown in figure 6 E-G.
**Reviewer #2**	
Fig. legends to 1C and 1D are swapped.	Corrected
Why is csw not necessary for PGC formation? It acts upstream of Ras. This is not discussed.	We now highlight this point in the text (and refer to studies on the sevenless kinase, which suggested a similar position of Csw parallel or downstream of Ras (page 6 line 107-).
Fig 3C. Consider changing the order of the ras-variants used: S35, G37, C40 instead of S35, C40, G37.	We changed the schematic in Figure 3C that should make the order of Ras

Review COMMONS

	variants more intuitive.
Fig 4A, B: Consider changing the order of the panels. Control, dp110-wt, dp110-CAAX, dp110-D954A, dp110-deltaRBD.	Order of constructs was rearranged
Fig S4 is mentioned in the text before S2 and S3. Consider changing the suppl. figure order.	Order of supplementary figures was rearranged
Page 12: Fig S1 A does not show PIP2 dynamics. Movie 1 is not available to this reviewer. The authors most likely refer to fig. S2.	Movie 1 was uploaded and figure calls were corrected
Page 13, 1st para: Why do the authors use glc heterozygous embryos to look at PIP3 and PIP2? Particularly so when they report later in the MS that glc+/- behave differently to wt controls in terms of PIP3 levels (Fig. 7C). By looking at gcl+/+, they might find that now PIP2 levels are different in gcl mutant embryos or that the differences between PIP3 levels in +/+ and -/- are larger than compared with +/-.	Since *gcl+/-* embryos form the same number of PGCs as WT but show a statistically significant increase in PI3K activity when comparing membrane to cytoplasm staining intensity, we favor using *gcl+/-* embryos, as these embryos may represent a more sensitive test for PIP2 and PIP3 levels.
Pages 15 and 16: revise figure calls in the text.	Figure calls were revised
M+M: How were gcl+/- and gcl-/- embryos identified?	Since all genetic manipulations in this alter the maternal contribution to the embryo, we us the term 'mutant' embryos referring to the maternal genotype (indicated on page 3 line 33 and more clearly stated in material and methods and reagent table). Embryos derived from mother of a specific maternal genotype are all identical, thus

	we can easily distinguish between embryos derived from homozygous mutant mothers (*gcl-/-*) or heterozygous mutant mothers (*gcl-/+)* In the reagents table we include the precise genotype description. “CyO” refers to the balancer chromosome commonly used to identify heterozygotes on the second chromosome. Flies with the CyO balancer have curly wings.
**Reviewer #3**	
Figure 1B: The authors describe that embryos with OptoSos still form buds which protruded from the cortex, but PGCs largely fail to cellularize (described in pg. 5). I'm not sure what they meant by "fail to cellularize" as this is not obvious to me when looking at the figure. The authors should describe how they know it's cellularized in the controls and not in the OptoSos or change the wording to "suggesting a failure to cellularize".	We used the word ‘protruded’ to describe our live observations. PGCs were quantified in fixed embryos, immunostained with anti-Vasa antibody to count Vasa positive cells (Fig 1C and D. We observe a lack of Vasa-positive PGCs, only in the light-activated OptoSos condition.
Fig. 1B, lines 4-5: at what stage are these embryos? Cycle 9? Cycle 14? Both?	Nuclear cycles of embryos for each panel are noted on the left side of each panel
Fig. 4A: add dp110-CAAX results to Results section	dp110-CAAX results are included in the Results section (p.9. line 177)
Figure 5C: The hyper-clustered phenotype they describe is hard to visualize in this figure (described in pg. 11). The authors should describe what is meant by "hyper-clustered".	We agree and re-worded the description of this

	observation to be clearer, page 11, line 226-.
Figure 7: When comparing Fig. 7A and 7B torsoHH/WK images, we can see that in Fig. 7A that PIP3 pattern changes such that PIP3 is now at the most posterior end where PGC will eventually form (compared to control that has low PIP3 in this region), but then in Fig. 7B they are looking at the buds and they say PIP3 levels decrease, which does not correspond to Fig. 7A. Are these simply different stages and PIP3 levels change over time (looking at Fig. 7C, PIP3 does not seem to change a lot over time)?	The figure legend now states more clearly that embryos were of different ages. We also explain in the text the apparent discrepancy in the patterns before and during budding (page13 line 266). The time points in figure 7C span nuclear cycle 10, not earlier (page14 line 274). By measuring membrane to cytoplasmic distribution, a more accurate comparison is possible at this stage.
p. 5, line 5: "Optosos" is written "OptoSos" elsewhere (suggest using OptoSos throughout)	Corrected
Is it possible that inhibition of myosin II recruitment is due to conversion of PIP2 -> PIP3, thus loss of PIP2, or is it that myosin is specifically recruited to regions where PIP2 is high? This seems like a point that should be added to the discussion.	This point is now discussed on page 20, line 403
p. 5, line 6: suggest adding a comma after "Ras" for clarity	Corrected
p. 5, last line: the genotype is "w^1118" (with ^ indicating a superscript), not "w^-1118", and is italicized (this should be corrected throughout)	Corrected
p. 6, line 2: replace "cellularizing" with "cellularization"	Corrected
p. 6, lines 11-13: Where is it shown that knockdown of csw, dsor1 and rolled did not restore PGC formation? The data are not present in Fig. 2C (could include in supp fig?)	We added these data as Supplementary figure 1
p. 7, line 1: replace "interfere" with "interferes"	Corrected
p. 7, last three lines: what is stated here, "Ras-G37 [activates] both the RalA and the PI3K pathways, and Ras-C40 activates the PI3K pathway" is not consistent with what	We apologize and corrected the figure

is diagrammed in Fig. 3C, where Ras-C40 is indicated as activating RalA (please correct either the text or the diagram)	
p. 11, lines 1-2: the Pi3K21B gene and transcript should be italicized (note that Pi3K21B is the official gene name on FlyBase)	Gene name was italicized
p. 11, lines 6-10: it might be helpful to explain how the p60 construct was overexpressed (current lines 9-10) before describing the results (current lines 7-8)	Clarification on p60 construct was added to p.11, line 215-
p. 12, paragraph 2, line 2: the PIP2 biosensor should be written as "PLCgamma[PH]:mCherry" throughout, not "PLCy[PH]:mCherry"; this should be changed in the figures as well as the text (Symbol font can be used to turn "g" into lower-case "gamma", both in Word and in Illustrator)	Gamma symbol was added
It would also be helpful to show the overlap of the PIP2 and PIP3 signals in control vs. gcl mutants at different stages so the relative distribution and intensity of the signals can be better appreciated (consider adding this as a supplementary figure).	Our data show that PIP2 is not affected by lack of GCL (Fig 6 B-D). We thus do not think that simultaneous imaging of PIP2 and PIP3 in *gcl-/-* would add to our conclusions. Furthermore, these experiments would require a significant time investment to generate the respective genotypes. Thus, we agree with the reviewer that this is experiment is beyond the scope of the paper.
p. 12, paragraph 2, line 3: it does not appear that the two PIP markers were used "simultaneously" in Fig. 6A; however, this is evident from Fig. S2 and Movie 1 (consider placing callouts to these earlier in the paragraph or moving the description of simultaneous expression and observation of the two markers later in the paragraph to avoid confusion)	We did simultaneously image PIP2 and PIP3 sensors and have added this as Movie 1 and also in supplementary Figure S4, which are now clearly referred to in the text.
p. 12, paragraph 2, line 7: replace "Fig. S1A" with "Fig. S2" (this was confusing)	Figure call was updated

p. 16: change "Fig. 7G-I" to "Fig. 8G-I"	Figure call was updated
p. 20, Deming reference: there appears to be a stray asterisk in the title	Asterisk was removed from reference
Fig. 1D: need to explain that the colors in the graph indicate the numbers of PGCs formed (this could also be added as a label across the top of the graph); in addition, the number of embryos examined for each genotype should be included in the legend	We added a label at the top of the graph and 'n' were added to figure legend
Fig. 2B: spell out where csw, dsor1 and rolled data are shown; also, "n" is not defined; was this the number of embryos per genotype?	We added these data as Supplemental Figure 1
Fig. 3B: "EV" should be defined in the legend; is this "empty vector"?	We are using a "-" to mark controls without transgene
Fig. 3C: see previous comment re: mistake in the diagram; I believe Ras-C40 was described as activating PI3K, not RalA	We apologize and corrected the figure
Fig. 4B, line 2: was the graph plotted from the data in panel (C) or panel (A)? panel (A) seems more likely, because the data in C is plotted in D; please correct the panel callout	Figure legend was updated to refer to the correct panel
Fig. 5C: describe "p60-TCEp3" in the legend	We added germplasm-targeting 3'UTR (TCEp3) to legend and the construct and reference are provided in Material and Methods section
Figure 6: In Fig. 6E-G, the "brightness" of PIP3 at the membrane corresponds to the images even with different views (posterior and orthogonal) and agrees with the graph. However, when looking at Fig. 6B, it looks to me that PIP2 is brighter in gcl+/-, but the opposite is true when looking at Fig. 6D (i.e., PIP2 looks brighter in gcl-/-). The authors might want to comment on this.	We have updated the figure to better reflect our observations.
Fig. 6A: define "(fire)" here or in the first figure legend where this is used	We added an inset for the fire lookup table to clearly define the pseudcolor scheme used in the image
Figure 8 title: "Actin fluorescence is increased in gcl-/- pole buds",But their graph in Fig. 8B comparing actin in gcl+/- to -/- is not significant	Thanks for catching our mistake, myosin not actin is changed
Fig. 8I: replace "Scarlett" with "Scarlet"	Corrected

Review COMMONS

Fig. 8D-F: Although the plots in panel E agree with the images in panel D, it is unclear why those in panel F are not more concordant. In F, myosin appears enriched at the cortex relative to the cytoplasm in gcl-/- mutants, which is hard to reconcile with the data in D-E.	We have updated the figure to better reflect our observations.
Fig. S2A: define the three time points shown here, and clarify that these are shown left to right (if this is indeed the case)	We removed S2A and updated the movie to replace it
Fig. S4: change "P60" to "p60" in the figure title	Corrected
Movie: The movies showing PIP2 and PIP3 in whole embryos are nice, but it would also be helpful to also include merged images of the two channels, so the reader can examine the relative accumulation of the two PIPs over time.	Merged images panel was added to the movie.

May 4, 2026

RE: JCB Manuscript #202604036T

Ruth Lehmann
Whitehead Institute for Biomedical Research

Dear Prof. Lehmann:

Thank you for submitting your revised manuscript entitled "GCL pruning of PIP3 establishes the soma - germline boundary" via Review Commons. Your paper was sent back to the original referees for re-review. They all now support publication so we would be happy to publish your paper in JCB pending final revisions necessary to meet our formatting guidelines (see details below).

In your final revision, please be sure to address the reviewers' final concerns in the manuscript text.

To avoid unnecessary delays in the acceptance and publication of your paper, please read the following information carefully.

A. MANUSCRIPT ORGANIZATION AND FORMATTING:

Full guidelines are available on our Instructions for Authors page, http://jcb.rupress.org/submission-guidelines#revised.
Submission of a paper that does not conform to JCB guidelines will delay the acceptance of your manuscript.

1) Text limits: Character count for Articles is < 40,000, not including spaces. Count includes abstract, introduction, results, discussion, and acknowledgments. Count does not include title page, figure legends, materials and methods, references, tables, or supplemental legends.

2) Figures limits: Articles may have up to 10 main text figures.

3) Figure formatting: Scale bars must be present on all microscopy images, including inset magnifications. Molecular weight or nucleic acid size markers must be included on all gel electrophoresis. Aspect ratios of images may not be altered.

**4) Statistical analysis: Error bars on graphic representations of numerical data must be clearly described in the figure legend. The number of independent data points (n) represented in a graph must be indicated in the legend.
**Statistical methods should be explained in full in the materials and methods. For figures presenting pooled data the statistical measure should be defined in the figure legends. Please also be sure to indicate the statistical tests used in each of your experiments (either in the figure legend itself or in a separate methods section) as well as the parameters of the test (for example, if you ran a t-test, please indicate if it was one- or two-sided, etc.). Also, if you used parametric tests, please indicate if the data distribution was tested for normality (and if so, how). If not, you must state something to the effect that "Data distribution was assumed to be normal but this was not formally tested."

**5) Abstract and title: The abstract should be no longer than 160 words and should communicate the significance of the paper for a general audience.
**Given that your abstract currently exceeds the 160 word limit, we kindly ask you to shorten the text a bit.
The title should be less than 100 characters including spaces. Make the title concise but accessible to a general readership.

6) Materials and methods: Should be comprehensive and not simply reference a previous publication for details on how an experiment was performed. Please provide full descriptions in the text for readers who may not have access to referenced manuscripts.

7) All antibodies, cell lines, animals, and tools used in the manuscript should be described in full, including accession numbers for materials available in a public repository such as the Resource Identification Portal. Please be sure to provide the sequences for all of your primers/oligos and RNAi constructs in the materials and methods. You must also indicate in the methods the source, species, and catalog numbers (where appropriate) for all of your antibodies. Please also indicate the acquisition and quantification methods for immunoblotting/western blots.

8) Microscope image acquisition: The following information must be provided about the acquisition and processing of images:
a. Make and model of microscope
b. Type, magnification, and numerical aperture of the objective lenses
c. Temperature
d. Imaging medium
e. Fluorochromes

f. Camera make and model
g. Acquisition software
h. Any software used for image processing subsequent to data acquisition. Please include details and types of operations involved (e.g., type of deconvolution, 3D reconstitutions, surface or volume rendering, gamma adjustments, etc.).

9) References: There is no limit to the number of references cited in a manuscript. References should be cited parenthetically in the text by author and year of publication. Abbreviate the names of journals according to PubMed.

10) Supplemental materials: There are strict limits on the allowable amount of supplemental data. Articles may have up to 5 supplemental figures. Please also note that tables, like figures, should be provided as individual, editable files. A summary of all supplemental material should appear at the end of the Materials and methods section.

**11) eTOC summary: A ~40-50-word summary that describes the context and significance of the findings for a general readership should be included on the title page. The statement should be written in the present tense and refer to the work in the third person.

12) Conflict of interest statement: JCB requires inclusion of a statement in the acknowledgements regarding competing financial interests. If no competing financial interests exist, please include the following statement: "The authors declare no competing financial interests." If competing interests are declared, please follow your statement of these competing interests with the following statement: "The authors declare no further competing financial interests."

13) ORCID IDs: ORCID IDs are unique identifiers allowing researchers to create a record of their various scholarly contributions in a single place. Please note that ORCID IDs are now *required* for all authors. At resubmission of your final files, please be sure to provide your ORCID ID and those of all co-authors.

14) A separate author contribution section following the Acknowledgments. All authors should be mentioned and designated by their full names. We encourage use of the CRediT nomenclature.

Please note that JCB now requires authors to submit Source Data used to generate figures containing gels and Western blots with all revised manuscripts. This Source Data consists of fully uncropped and unprocessed images for each gel/blot displayed in the main and supplemental figures. For assays performed using capillary electrophoresis and/or immunoassay-based detection, authors should instead provide the electropherogram graph(s) for each experiment, plotting fluorescence/chemiluminescence intensity vs. molecular weight/size. Please be sure to provide one Source Data file for each figure gels, blots, and/or capillary electrophoresis assays along with your revised manuscript files. File names for Source Data figures should be alphanumeric without any spaces or special characters (i.e., SourceDataF#, where F# refers to the associated main figure number or SourceDataFS# for those associated with Supplementary figures). For traditional gels and blots, the lanes of the gels/blots should be labeled as they are in the associated figure, the place where cropping was applied should be marked (with a box), and molecular weight/size standards should be labeled wherever possible. For capillary electrophoresis assays, each trace in the graph should be color-coded and labeled to indicate which protein, gene, or sample is being measured (please try to avoid red/green combinations to accommodate our color-blind readers).

Source Data files will be made available to reviewers during evaluation of revised manuscripts and, if your paper is eventually published in JCB, the files will be directly linked to specific figures in the published article.

Source Data Figures should be provided as individual PDF files (one file per figure). Authors should endeavor to retain a minimum resolution of 300 dpi or pixels per inch. Please review our instructions for export from Photoshop, Illustrator, and PowerPoint here: https://rupress.org/jcb/pages/submission-guidelines#revised

Journal of Cell Biology now requires a data availability statement for all research article submissions. These statements will be published in the article directly above the Acknowledgments. The statement should address all data underlying the research presented in the manuscript. Please visit the JCB instructions for authors for guidelines and examples of statements at (https://rupress.org/jcb/pages/editorial-policies#data-availability-statement).

B. FINAL FILES:

Please upload the following materials to our online submission system. These items are required prior to acceptance. If you have any questions, contact JCB's Managing Editor, Lindsey Hollander (lhollander@rockefeller.edu).

-- An editable version of the final text (.DOC or .DOCX) is needed for copyediting (no PDFs).

-- High-resolution figure and MP4 video files: See our detailed guidelines for preparing your production-ready images, https://jcb.rupress.org/fig-vid-guidelines.

-- Cover images: If you have any striking images related to this story, we would be happy to consider them for inclusion on the journal cover. Submitted images may also be chosen for highlighting on the journal table of contents or JCB homepage carousel. Images should be uploaded as TIFF or EPS files and must be at least 300 dpi resolution.

It is JCB policy that if requested, original data images must be made available to the editors. Failure to provide original images upon request will result in unavoidable delays in publication. Please ensure that you have access to all original data images prior to final submission.

The license to publish form must be signed before your manuscript can be sent to production. A link to the license to publish form will be sent to the corresponding author only. Please take a moment to check your funder requirements before choosing the appropriate license.

Additionally, JCB encourages authors to submit a short video summary of their work. These videos are intended to convey the main messages of the study to a non-specialist, scientific audience. Think of them as an extended version of your abstract, or a short poster presentation. We encourage first authors to present the results to increase their visibility. The videos will be shared on social media to promote your work. For more detailed guidelines and tips on preparing your video, please visit https://rupress.org/jcb/pages/submission-guidelines#videoSummaries.

Thank you for your attention to these final processing requirements. Please revise and format the manuscript and upload materials within 14 days. If you need an extension for whatever reason, please let us know and we can work with you to determine a suitable revision period.

Please contact the journal office with any questions at cellbio@rockefeller.edu.

Thank you for this interesting contribution, we look forward to publishing your paper in Journal of Cell Biology.

Sincerely,

Julius Brennecke
Monitoring Editor
Journal of Cell Biology

Gabriele Stephan
Scientific Editor
Journal of Cell Biology

Reviewer #1:

The authors answered nicely to all our minor comments on this exciting manuscript.
we noted some minor typos remaining:
p.11, l.206 (Fig.S4A-B) should be replaced by (Fig.S3A-B)
p.12, l.245 (Fig.S3A-B) should be replaced by (Fig.S4A-B)

Reviewer #2:

With one exception (see below), the authors have addressed satisfactorily most of my comments. The manuscript is even better than the original.
The only matter that I would advise the authors to consider is the clarification of the meaning of the glc-/- and +/- genotypes. I appreciate the authors reminding me that CyO carries a dominant mutation that produces curly wings (how could I have missed that after 40 years working with flies??). However, the nomenclature -and its explanation in the Reagents section- is, in my opinion, still confusing. The text, (page 3, line 33), states "gcl-/- embryos (referring to the maternal genotype)" while the relevant section in the Reagents List indicates "gclΔ/CyO (referred to as gcl-/- in text)". As I understand it, gclDelta/CyO females would produce oocytes containing maternally provided Glc. Hence and following the authors' nomenclature, these would correspond to +/- embryos rather than -/-.

I would suggest the authors 1) to clarify the fact that gcl-/- embryos are derived from mutant glc mothers and that +/- are laid by heterozygous mothers (this is not referred to in the text). 2) Explain in the M+M section (where the generation of gcl embryos is not described) that the paternal phenotype does not influence the PGC budding phenotype. 3) Revise the Reagents List to make clear that gclDelta/CyO mothers lay gcl+/- embryos, not -/-. Perhaps the use of gcl mat-/- embryos versus gcl mat+/- embryos improves clarity?

Reviewer #3:

Summary:
Although Torso is known to antagonize primordial germ cell (PGC) formation, the underlying mechanisms remain unclear. Canonical Torso signalling typically results in activation of Ras. However, the authors show that Ras-mediated suppression of PGC formation is independent of the Raf/MEK/ERK pathway. Instead, they uncover an unexpected role for Torso in activating phosphoinositide 3-kinase (PI3K) that promotes formation of PIP3 enriched posterior membrane domains. The resulting increase in PI3K activity disrupts PGC formation. Furthermore, they show that by promoting Torso degradation, the ubiquitin ligase adaptor Germ Cell-Less (GCL) primes the posterior membrane with reduced PIP3 to facilitate PGC formation. Lastly, the authors suggest a model where antagonistic relationship between GCL and Torso influences actomyosin contractility that may allow the bud to constrict for proper PGC formation.

The authors appear to have thoroughly addressed the comments of all the reviewers, and the clarity of the manuscript and figures are much improved, including the description of the effect of wild-type p60 on PGC formation (lines 219-225) and addition of the diagrams in Figs 4 and 5. One omission I noticed on rereading the Discussion is that the pioneering work on mz Pten mutants in Drosophila embryogenesis and pole cell formation (PMID: 15743877) was neither cited nor discussed. Because most of the references related to Pten are from Dictyostelium, which can divide by cytofission as well as actomyosin-mediated contractile ring formation, the Drosophila Pten data could provide more relevant support for the authors' conclusions (bottom of p. 18 to top of p. 19).

I have only some small comments (below) that could improve the writing.

Minor corrections:

line 22: could delete "The" before constriction (awkward)
line 33: description of maternal genotype in this instance is clear, but could convey better the idea that paternal genotype has no effect; also, as written, this information conflicts with information provided in the Reagents List, which states that gcl-Delta/CyO is referred to as gcl-/- in the text; the genetics and genotypes should be clarified for the reader
line 57: could delete "the" and "pathway" ("endocytosis" is sufficient)
line 105: should be "act", not "acts"
line 111: could delete "the" before "activation (unnecessary)
line 113: could delete "the" before "transcriptional activation" (ditto)
line 126: could delete "the" before "constitutive" (ditto)
line 139: do the authors mean to say "separation-of-function" mutants?
line 202: could delete "the" before "plasma membrane
line 206: should be Fig. S3A-B (rather than Fig. S4A-B); "p60" is not capitalized
line 214: should be "inhibit", not "inhibits" ("SH2 domains" is plural)
line 245: should be Fig. S4A-B (rather than Fig. S3A-B)
line 293: could add a comma and "which" after parenthesis to avoid repetition

Response to reviewers:
We thank the reviewer for their careful reading and helpful comments. We have made all suggested changes and indicated them in red below.

Reviewer #1:

The authors answered nicely to all our minor comments on this exciting manuscript.
we noted some minor typos remaining:
p.11, l.206 (Fig.S4A-B) should be replaced by (Fig.S3A-B)
p.12, l.245 (Fig.S3A-B) should be replaced by (Fig.S4A-B)

We are so sorry about the oversight. We thank the reviewer for pointing this out. It has been corrected.

Reviewer #2:

With one exception (see below), the authors have addressed satisfactorily most of my comments. The manuscript is even better than the original.
The only matter that I would advise the authors to consider is the clarification of the meaning of the glc-/- and +/- genotypes. I appreciate the authors reminding me that CyO carries a dominant mutation that produces curly wings (how could I have missed that after 40 years working with flies??). However, the nomenclature -and its explanation in the Reagents section- is, in my opinion, still confusing. The text, (page 3, line 33), states "gcl-/- embryos (referring to the maternal genotype)" while the relevant section in the Reagents List indicates "gclΔ/CyO (referred to as gcl-/- in text)". As I understand it, gclDelta/CyO females would produce oocytes containing maternally provided Glc. Hence and following the authors' nomenclature, these would correspond to +/- embryos rather than -/-.

I would suggest the authors 1) to clarify the fact that gcl-/- embryos are derived from mutant glc mothers and that +/- are laid by heterozygous mothers (this is not referred to in the text). 2) Explain in the M+M section (where the generation of gcl embryos is not described) that the paternal phenotype does not influence the PGC budding phenotype. 3) Revise the Reagents List to make clear that gclDelta/CyO mothers lay gcl+/- embryos, not -/-. Perhaps the use of gcl mat-/- embryos versus gcl mat+/- embryos improves clarity?
We corrected the reagent table and apologize for the confusion. We did not mean to insult this fly researcher, but must have misunderstood their comment. In Materials and Methods, section D. melanogaster Maintenance and Genetics, we added a sentence that clearly explains our nomenclature for *gcl-/-* and *gcl-/+* embryos. Since we only deal with maternally inherited factors

in this study, we kept the simple *gcl-/-* and *gcl-/+* nomenclature in the text. However, to avoid any confusion when analyzing the data, we now clearly outline maternal genotypes and mode of inheritance in the figure legends.

Reviewer #3:

Summary:
Although Torso is known to antagonize primordial germ cell (PGC) formation, the underlying mechanisms remain unclear. Canonical Torso signalling typically results in activation of Ras. However, the authors show that Ras-mediated suppression of PGC formation is independent of the Raf/MEK/ERK pathway. Instead, they uncover an unexpected role for Torso in activating phosphoinositide 3-kinase (PI3K) that promotes formation of PIP3 enriched posterior membrane domains. The resulting increase in PI3K activity disrupts PGC formation. Furthermore, they show that by promoting Torso degradation, the ubiquitin ligase adaptor Germ Cell-Less (GCL) primes the posterior membrane with reduced PIP3 to facilitate PGC formation. Lastly, the authors suggest a model where antagonistic relationship between GCL and Torso influences actomyosin contractility that may allow the bud to constrict for proper PGC formation.

The authors appear to have thoroughly addressed the comments of all the reviewers, and the clarity of the manuscript and figures are much improved, including the description of the effect of wild-type p60 on PGC formation (lines 219-225) and addition of the diagrams in Figs 4 and 5. One omission I noticed on rereading the Discussion is that the pioneering work on mz Pten mutants in Drosophila embryogenesis and pole cell formation (PMID: 15743877) was neither cited nor discussed. Because most of the references related to Pten are from Dictyostelium, which can divide by cytofission as well as actomyosin-mediated contractile ring formation, the Drosophila Pten data could provide more relevant support for the authors' conclusions (bottom of p. 18 to top of p. 19).
In the referenced study by the Wodarz lab, the authors observed a lack of pole cells due to failure of germ plasm assembly during oogenesis and of nuclear migration during early embryogenesis. Thus, they found a role for PTEN that precedes its role in the physical process of pole cell formation that occurs after nuclei migrated into the germplasm and is the focus of our study. Indeed, this earlier role of PTEN during oogenesis obscured its later role in pole cell formation, described in our study. In response to the reviewer's comment and to avoid confusion, we now mention this study in the discussion (line 384).

I have only some small comments (below) that could improve the writing.

Minor corrections:
We followed this reviewer's grammatical suggestions and made changes.
line 22: could delete "The" before constriction (awkward)

line 33: description of maternal genotype in this instance is clear, but could convey better the idea that paternal genotype has no effect; also, as written, this information conflicts with information provided in the Reagents List, which states that gcl-Delta/CyO is referred to as gcl-/- in the text; the genetics and genotypes should be clarified for the reader

This has been clarified, in particular maternal inheritance patterns are now clearly noted in all figure legends, see also our comments to reviewer 2.

line 57: could delete "the" and "pathway" ("endocytosis" is sufficient)

line 105: should be "act", not "acts"

line 111: could delete "the" before "activation (unnecessary)

line 113: could delete "the" before "transcriptional activation" (ditto)

line 126: could delete "the" before "constitutive" (ditto)

line 139: do the authors mean to say "separation-of-function" mutants?

line 202: could delete "the" before "plasma membrane

line 206: should be Fig. S3A-B (rather than Fig. S4A-B); "p60" is not capitalized

we corrected, see comment to reviewer 1

line 214: should be "inhibit", not "inhibits" ("SH2 domains" is plural)

line 245: should be Fig. S4A-B (rather than Fig. S3A-B)

line 293: could add a comma and "which" after parenthesis to avoid repetition
